# Feasibility study to identify women of childbearing age at risk of pregnancy not using any contraception in The Health Improvement Network (THIN) database

**DOI:** 10.1186/s12911-020-01184-0

**Published:** 2020-07-18

**Authors:** Lucía Cea Soriano, Alex Asiimwe, Mieke Van Hemelrijck, Cecilia Bosco, Luis A. García Rodríguez

**Affiliations:** 1 Spanish Centre for Pharmacoepidemiologic Research (CEIFE), Madrid, Spain; 2 Department of Public Health and Maternal and Child Health. Faculty of Medicine, Universidad Complutense de Madrid, Madrid, Spain; 3 Bayer AG, Berlin, Germany; 4 King’s College London, Translational Oncology & Urology Research (TOUR), London, UK

**Keywords:** Database, Pregnancy, Feasibility, Contraception, THIN

## Abstract

**Background:**

Worldwide the rate of unplanned pregnancies is more than 40%. Identifying women at risk of pregnancy can help prevent negative outcomes and also reduce healthcare costs of potential complications. It can also allow the investigation of the natural history of pregnancy outcomes, such as ectopic pregnancies or miscarriages. The use of medical records databases has been a crucial development in the field of pharmacoepidemiology – e.g. The Health Improvement Network (THIN) database is a validated database representative of the UK population. This project aimed to test the feasibility of identifying a population of women of childbearing age who are at risk of pregnancy not using any contraception in THIN database.

**Methods:**

First a cohort of women of childbearing age (15-45yo) was identified. By applying a computer-based algorithm, containing codes for contraception methods or other suggestion of contraception, the risk of pregnancy was then ascertained. Next, two validation steps were implemented: 1) Revision of medical records/free text and 2) Questionnaires were sent to primary care practitioners (PCP) of women whose medical records had been reviewed. Positive predicted values (PPV) were calculated.

**Results:**

A total of 266,433 women were identified in THIN. For the first validation step, 123 records were reviewed, with a PPV of 99.2% (95%CI: 95.5–99.9). For the questionnaires step, the PPV was of 82.3% (95%CI: 70–91.1). Information on sexual behaviour and attitudes towards conception was not captured by THIN.

**Conclusion:**

This study shows that by applying a comprehensive computer-based algorithm, THIN can be used to identify women at risk of pregnancy.

## Background

Between 2010 and 2014, worldwide, approximately 44% of pregnancies were unintended. However, there are regional variations. For instance, the proportion of unintended pregnancies in the Latin American region and in Southern Africa were 69 and 66%, respectively. This proportion was 29% in Northern Europe. More than 50% of these pregnancies ended in abortion, representing a healthcare burden due to the procedure itself, medical complications, psychological impact and the concomitant costs [1]. Part of these unintended pregnancies are a result of women not using contraception methods. Identifying these women may help reduce the proportion of unintended pregnancies and potential complications. Furthermore, if women are not using any contraception method and pregnancy occurs, the natural history of pregnancy outcomes can be investigated. One relatively inexpensive method to identify people at risk of several conditions is the use of health care databases.

Health care databases are a standard source of information for post-approval pharmacoepidemiologic studies as well as the natural history of selected disorders [2–4]. They provide prospectively collected information for large populations and allow the study of multiple outcomes, including rare or long-term consequences of drug use. In the context of pregnancies, the main limitations of healthcare databases are that neither pregnancy nor pregnancy outcomes are routinely recorded in most administrative databases, and that important reproductive information may not be always available [5]. The Health Improvement Network (THIN) has been previously validated for pharmacoepidemiologic research and provides: i) longitudinal data of a large stable population, ii) detailed and unbiased prospective assessment of prescriptions, iii) centralised primary care provider records with clinical information that includes obstetric notes for each pregnancy, iv) linkable birth information, and v) long-term follow up data for infants [2].

The aim of this study was to develop and validate a methodology to identify “women who are at risk of pregnancy not using any contraception method” in the THIN database. Moreover, we assessed whether information on important factors of history of sexual behaviour could be retrieved through questionnaires or/and medical records (validation step) via free text comments that are not routinely captured in THIN.

## Methods

### Setting

Using the UK THIN database, which has been described in detail previously [2], we conducted a cross-sectional study. Briefly, the information in THIN is recorded systematically by primary care providers (PCPs). Diagnoses and procedures are recorded using the *Read* classification, and prescriptions for drugs and devices are coded using a drug dictionary Gemstrip, based on the MULTILEX classification [6, 7]. Prescriptions ordered by the PCPs are recorded automatically in the database. In the UK, PCPs centralize the prescription of drugs to their patients. The maternity care provided by the National Health System includes PCPs, specialists, and hospitals. Primary care practitioners typically continue the care of their patients during pregnancy, working together with nurses and midwives at their practices; all of them record the information in THIN. PCPs may also record further details observations or notes in free-text comments. This information is only available on request and is anonymised to ensure patient privacy.

### Study cohort

First, a cohort of women of childbearing age, aged 15–45 years old, between September 2016 and December 2016 (enrolment period) with a registration status of permanent at the time of last available update of THIN was identified. During the study period, September through December 2016, women aged 15—45 years with a registration status of permanent at the time of last available update of THIN and at least 1-year enrolment with a primary care physician (PCP) were enrolled in the study. Once the study population was identified, women at risk of pregnancy were ascertained.

### Ascertainment of at risk of pregnancy not using any contraception

Using Stata package version 12.0 (StataCorp LP, College Station, TX, USA) a computer-based algorithm was developed to identify women at risk of pregnancy not using any contraception method. The algorithm used Read codes (diagnostic dictionary) or/and Gemstrip codes (drug dictionary) to detect and exclude all women with one of the following conditions suggestive of not being at risk of pregnancy (i.e. exclusion criteria):

- Read codes suggestive of infertility and subfertility (primary and iatrogenic) any time prior to and during the study enrolment (Additional files 1 and 2)
- Read code of menopause (Additional file 3)
- Read and Gemstrip codes of prescribed contraceptive methods in the year prior to and during the enrolment to the study. For long-acting reversible contraceptives (LARCs), natural life cycle was considered that is we looked within the 5 years prior to the study for Cu-IUDs and LNG-IUS and in the previous 3 years for progesterone -only implants (Additional file 4).
- Read codes of non-prescribed contraception such as condoms, rhythmic method or read codes suggestive of sexual inactivity in the year prior to and during the enrolment to the study (Additional file 5).
- Read codes suggestive of partner vasectomy within the any time prior to and during the study enrolment (Additional file 7).
- Read codes suggestive of pregnancy within the 6 months prior to and during the study enrolment (Additional file 6). Six-month period is to increase the sensitivity of the algorithm in enrolling women who are at risk of pregnancy during the study period.

Women were classified as at risk of pregnancy if they were free of all the conditions above.

### Validation

In order to quantify the extent of potential misclassification of the cohort of women at risk of pregnancy not using any contraception, we carried out two independent validation steps.

#### Validation step 1: medical records/free text comments

First, stratified random sampling was performed to select 150 women from THIN collaborating practices. An equal number of individuals (*N* = 25) was allocated to the following age intervals groups: 18–20, 20–25, 25–30, 30–35, 35–40, and 40–45. Next, medical records/free text comments assigned to specific Read codes suggestive of gynaecological conditions or contraceptive management available in the enrolment period and one-year prior for these 150 women were requested. Then the medical records of women were manually reviewed after incorporating free text comments. Based on this review women were classified as follows:

- Confirmed case: All women assigned to be at risk of pregnancy
- Non case: All women assigned not to be at risk of pregnancy

#### Validation step 2: questionnaires

Subsequently, questionnaires (Additional file 8) were sent to the PCPs of the same random sample of women. The questionnaires inquired information on women’s sexual activity, use of contraceptive and contraceptive methods (if any). The following information was specifically defined:

i) Pregnancy at the time of filling the questionnaire
ii) Contraception use in two different time frames; time of filling out the questionnaire and September–December 2016
iii) Type of contraception among users (e.g. oral contraception, LARCs, condoms, calendar methods, partner vasectomy)
iv) Pregnancy intention
v) Sexual activity

As recall bias was likely to be present when answering questions related to events occurring about 1 year earlier at the time of completing questionnaire, two time frames were used: 1) time of filling out the questionnaire (first trimester 2018), and 2) the enrolment period (September–December 2016). The first timeframe was not likely to be affected by recall compared to the second timeframe.

Considering that the “unknown” option in the questionnaire could mean either at risk or not, two definitions were established for analytical purposes: “unknown” considered as a non-case and “unknown” considered as a confirmed case and therefore excluded from the analysis.

### Data analysis

In both validation stages, positive predictive values (PPVs) of the condition (being at risk of pregnancy not using any contraception) were calculated. Free text comments and questionnaire information was used as gold standard.

To describe the performance of the developed algorithm, PPV was estimated as the proportion of “confirmed cases” identified in the validation cohort by the algorithm that were determined to be so by the gold standard (either free text comments and/or questionnaires). The PPV was calculated as using the numerator the number of women identified being at risk of pregnancy not using contraception through medical records/free text comment in validation step 1 and questionnaires using two different time frames in validation step 2 (questionnaires), separately divided by number of women identified being at risk of pregnancy not using contraception by algorithm. The corresponding exact two-sided 95% confidence interval (CI) for the PPV was calculated.

Since two different validation steps were carried out, a crosslink step between medical records/free text comments and PCP questionnaires was performed. Information reported in the questionnaires was used as gold standard to estimate the percentage agreement between the information contained in medical records/free text comments. Percentage agreement was calculated by dividing the number of women confirmed as cases or non-cases by both validation methods over the total number of women with available information.

For the first validation step, PPV was calculated for all women in the sub cohort. All the PPVs for the validation step 2 involving questionnaire were calculated with PCP questionnaire. For the second validation step and all other analysis that involved questionnaire information where missing information was expected, analysis was performed only on PCP with available information. There was no imputation for missing data.

## Results

### Study cohort

**Fig. 1:**
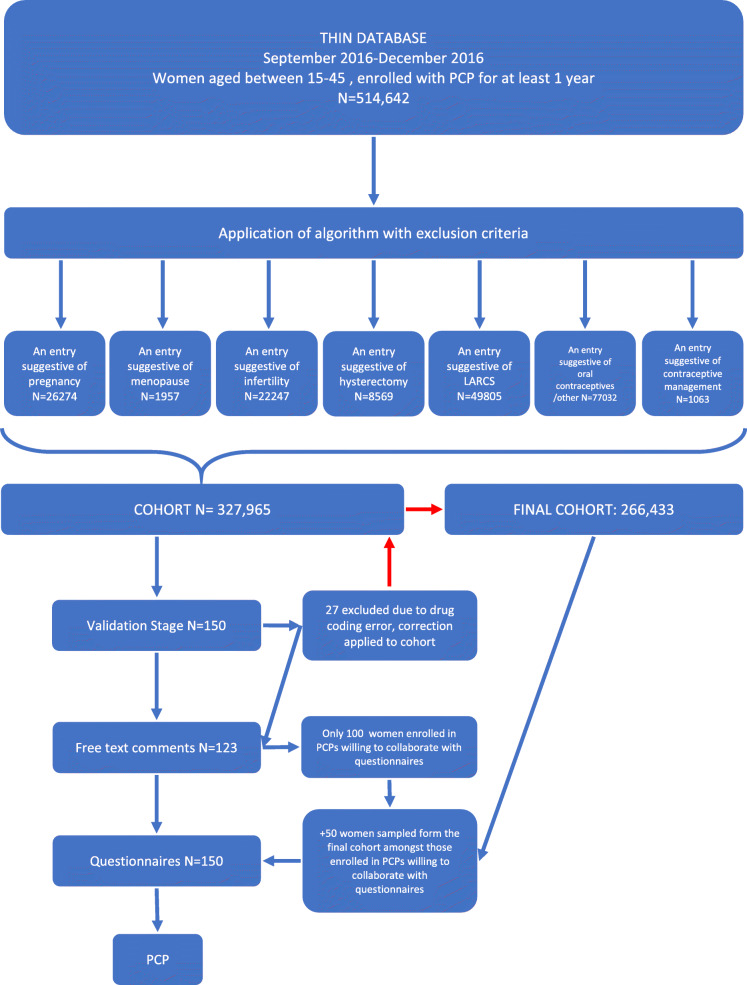
Flow chart showing participants selection and validation steps

During the study period based on the inclusion criteria of age and being registered with a PCP for at least 1 year, 514,642 women were identified. After applying the algorithm, 186,947 meeting one of the exclusion criteria were excluded. Therefore, a total of 327,695 women at risk of pregnancy were included in the initial study cohort. However, after identification of missing drug codes the final cohort consisted on 266,433 women. A flowchart of the patients selection process can be found in Fig. 1.

### Validation steps

#### Medical records and free text comments

Medical records were obtained from 150 women. At the time of the request of medical records, all 150 were considered to be women at risk of pregnancy. In between we discovered that certain drug codes had been missing from the search list used to remove women not at risk of pregnancy. When we included those additional drug codes, 27 (17%) were not considered anymore at risk of pregnancy. Therefore, 123 were eligible women at risk of pregnancy for whom medical records were reviewed. Out of these 123 women, only 8 (6.5%) had at least one free text comment inserted in the medical records, while remaining ones did not include any extra information. After reviewing the medical records, for 122 women there was no suggestion that they were not at risk of pregnancy. Only one woman was on LARCs 3 years prior to last quarter 2016 and our algorithm did not capture this case. That resulted in a PPV of 122/123 (99.2% (95% CI: 95.5–99.9)) when we used the entire sample of women at risk of pregnancy. However, amongst those with free text, the PPV was 87.5% (7/8) (95%CI: 52.9–97.8).

#### Questionnaires

Among the eligible set of 123 for whom medical records were obtained, 100 women (81.3%) were enrolled in practices that were willing to collaborate with questionnaires. An additional 50 women (for whom medical records were not obtained) were sampled from the remaining cohort enrolled amongst in collaborating practices.

A total of 133/150 questionnaires were returned (response rate of 88.7%: however, 5 questionnaires were sent empty resulting in a response rate of 85.3%). Table 1 below shows the summary of questionnaires results.

**Table 1:** Main items and characteristics reported in the questionnaires sent to PCPs

Questionnaires sent to PCPs *N* = 150	N	%
Questionnaires returned	**133**	
Empty	**5**	**3.8**
Valid	**128**	**95.2**
VALID	**128**	**%**
Confirmation of using contraception bet Sept-Dec 2016		
No	51	39.8
Unknown	66	51.6
Yes	11	8.6
LARCs	4	36.4
Oral contraception	3	27.3
Sterilization	1	9.1
Barrier	3	27.3
Items at the time of filling in the questionnaire: Feb 2018	**128**	**%**
Confirmation of using contraception at the time of filling questionnaire		
No	46	35.9
Unknown/empty	60	46.9
Yes	22	17.2
Oral contraception	11	50.0
Condoms/barrier	2	9.1
Implant (LARCs)	8	36.3
Sterilization	1	4.5
Ever use of contraception (any time prior sept 2016)	**128**	**%**
no	16	12.5
Unknown/Empty	70	54.7
Yes	42	32.8
Oral contraception	23	54.7
Condoms/barrier	4	9.5
LARCs	8	19.0
Ring	1	2.4
Empty/unspecified	6	14.3
Confirmation of pregnancy	**128**	**%**
No	97	75.8
Unknown	27	21.1
Yes	4	3.1
Wiliness of becoming pregnant	**128**	**%**
No	25	19.5
Unknown/Empty	97	75.8
Yes	6	4.7
Trying to conceive	**128**	**%**
No	28	21.9
Unknown/Empty	96	75.0
Yes	4	3.1

#### Results at the time of computer assignment (enrolment period)

In the set of returned questionnaires (*N* = 128), 39.8% of women (*N* = 51) were reported by the PCP as not using any contraception, 11 women (8,6%) were reported by the PCP as using contraception and for 66 women (51.6%) the PCP did not provide feedback. Out of these 11 women using contraception, 1 woman had the implant inserted 3 years prior to last quarter of 2016 and our algorithm did not capture this woman (same woman than the one found in medical records), 2 were reported to be on COC but no prescriptions were recorded in the medical records, another one underwent sterilization, another was using a progestogen and other women was under COC, three other women reported to use LARCs and three women were using barrier methods, unrecorded in the database. When excluding the unknown data (*N* = 66), we obtained a PPV of 82.3% (95% CI:0.70–0.91) (51/62).

#### Results from time period when filling out the questionnaire

The corresponding data for the time period when filling out the questionnaire were as follows: 35.9% of women were reported by the PCP as not using contraception, 17.2% of women were reported by the PCP as using contraception (50% on oral contraception) and in 46.9% the PCP did not provide feedback. Among women not using any kind of contraception (*N* = 46), 21 (45.6%) women had ever used contraceptive methods (71% oral contraception), 28.2% never used contraceptive methods and 22.7% were unknown.

At the time of filling the questionnaires only four women were pregnant (3.1%), a total of 25 women (19.5%) were not willing to get pregnant based on the feedback from the PCP and 75.8% the PCP did not provide feedback on this question. PCPs reported that 4 (3.1%) women were currently (first trimester 2018) trying to conceive, a total of 28 (21.9%) women were not and PCP responded unknown in 75.0% of women. When excluding the unknown data (*N* = 60), we obtained a PPV of 68.6% (95% CI:0.55–0.78) (46/68).

#### Cross link between medical records/free text comments and PCP questionnaires

Among the set of women present in both validation exercises, analyses of medical records/free-text comments and questionnaires were compared. The proportion of confirmed/non confirmed cases across each validation exercise is shown in Table 2 and Table 3. It should be noted that focus was only on the variable of case status (i.e. nor using any contraception at the time of study entry) and no other characteristics that were only requested in the questionnaire. There was a total of 82 women with information in both steps.

**Table 2:** Cross link among both validation exercises

Free text comments	PCP questionnaire Gold Standard		
	Confirmed Cases	Non confirmed cases	Total
Confirmed cases	33	48	81
Non confirmed cases	0	1	1
Total	33	49	82

**Table 3:** Cross link among both validation exercises excluding unknown cases from questionnaires

Free text comments	PCP questionnaire Gold Standard		
	Confirmed Cases	Non confirmed cases	Total
Confirmed cases	33	6	39
Non confirmed cases	0	1	1
Total	33	7	40

When using “definition a”, the PPV of free text comments was 40.7% (95% CI:29.9–52.2). When excluding unconfirmed cases from questionnaires (“definition b”), the PPV of free text comments was 84.6% (95% CI:69.5–94.1) and NPV was 100%.

## Discussion

The present study’s results showed that the algorithm used to identify women at risk of pregnancy had a PPV of 99.2% when compared to medical records/free text. When looking at free text alone the PPV was of 87.5%. The low number of records with free text comments difficults the generalization of the last results. Regarding the questionnaires, results showed PPVs of 82.3% and of 68.6% at the time of and at the time of filling up the questionnaires respectively. Therefore, our results show that the algorithm can accurately identify and retrieve a high proportion of women at risk of getting pregnant when compared to information contained in medical records/free text comments and specific questionnaires. This implies that medical record revision and questionnaires can be avoided cutting costs and research time.

As previously motioned, worldwide 4/10 pregnancies are unplanned. As a result, essential health interventions provided once a woman and her partner decide to have a child will be too late in 40% of pregnancies [8]. Periconceptional care is key for the health of the baby and mother. For example, maternal undernutrition and iron-deficiency anaemia increase the risk of maternal death, accounting for at least 20% of maternal mortality worldwide. Prevention and detection of infectious diseases and chronic diseases (i.e., HIV, tetanus, arterial hypertension, diabetes) can also help improve pregnancy outcomes. Other factors are the psychosocial effects of unplanned pregnancies where, for instance, violence against pregnant women can result in premature delivery and low-weight infants. Drug-addiction disorders (smoking, alcohol and usage of other drugs) can also increase the risk of severe health outcomes that affect both mothers and babies [8].

Identifying women at risk of pregnancy can also help prevent the potential teratogenic effects of drugs used to treat other conditions women may present. For instance, some drugs used in the treatment of autoimmune diseases (i.e. methotrexate) are contraindicated during pregnancy [9]. Furthermore, it has been shown that extreme maternal ages can have a negative impact in pregnancy outcomes. In a recent study maternal age over 40 years was found to be an independent risk factor for preterm delivery, gestational diabetes mellitus, caesarean section and abnormal foetal presentation amongst other [10].

Another important consequence of unplanned pregnancies are the costs to healthcare systems. In 2010 the U.S. government expenditures on births, abortions and miscarriages resulting from unintended pregnancies nationwide totalled U$D 21 billion [11]. Therefore, the identification of women at risk of pregnancy can help PCPs to implement promotive, preventive and curative health interventions that are effective in in improving maternal and child health.

A strength of the present study is the use of the largest electronic medical record databases in primary care setting worldwide. The validity of this database has been demonstrated in previous studies [12–15]. Patients included in THIN database are representative of the entire UK population with respect to age, sex and geographical region [16]. Hence, results may be extrapolated to the general UK population. Further strengths are the validation steps provided us with the information on whether contacting PCPs can add additional information on important sexual history not coded in THIN. As with all studies using medical records, the reliability of the results is dependent on the quality and completeness of the recording of patient data representing a potential limitation. Ascertainment of women at risk of pregnancy not on contraception may not have identified all, as some women may attend a setting outside their general practice to receive contraceptives, procedures to undergo sterilization etc. In that instance, any information not transferred to their PCP would not have been captured in THIN database. However, the proposed validation step using questionnaires (validation step 2) served to estimate the extent of under recording in THIN. A potential limitation of this validation method is that PCPs have access to other secondary care such as letters form specialist, family planning which are not systematically and directly captured in the THIN database, and maybe, in a few instances, they have recollection of some information not entered in any document. Another limitation is the high proportion of PCP’s that did not know if women were at risk of pregnancy. Due to data anonymization, the reasons for this escape the reach of our researchers.

## Conclusion

The present study results showed that THIN database can be used to identify women at risk of pregnancy and build a cohort for future studies. For instance, once women at risk of pregnancy not using recordable contraception methods are identified they can be followed up in order to understand the natural history of ectopic pregnancy, miscarriages, preterm births amongst other pregnancy outcomes. However, information on sexual behaviour and attitudes towards conception cannot be captured by THIN, thus this database is not an accurate source of information for these purposes.

## Supplementary information

**Additional file 1.** Read Code suggestive of iatrogenic infertility and subfertility. List of Read codes.

**Additional file 2.** Read Code suggestive of infertility and subfertility. List of Read codes.

**Additional file 3.** Read Code suggestive of menopause. List of Read codes.

**Additional file 4.** Read codes suggestive of long acting reversible contraception (LARC). List of Read codes.

**Additional file 5.** Read codes suggestive of other ways of contraception. List of Read codes.

**Additional file 6.** Read codes suggestive of pregnancy. List of Read codes.

**Additional file 7.** Read codes suggestive of vasectomy. List of Read codes.

**Additional file 8.** Questionnaire sent to the PCP. List of Read codes.

## Data Availability

Data will be available upon request.
