# Supplementary material for: Feasibility study to identify women of childbearing age at risk of pregnancy not using any contraception in The Health Improvement Network (THIN) database
Source: BMC Med Inform Decis Mak. 2020 Jul 18;20:164. doi: 10.1186/s12911-020-01184-0 (PMC7368731; doi:10.1186/s12911-020-01184-0)
Supplement: Supplementary file 1 — Additional file 1. Read Code suggestive of iatrogenic infertility and subfertility. List of Read codes. [file 12911_2020_1184_MOESM1_ESM.docx]

# Appendix 1. Read Code suggestive of iatrogenic infertility and subfertility

| **Read code** | **Descriptor** |
| --- | --- |
| 7E04400 | Subtotal abdominal hysterectomy |
| 7E04E00 | Laparoscopic subtotal hysterectomy |
| 7E04F00 | Subtotal abdominal hysterectomy with conservation of ovaries |
| 7E04300 | Total abdominal hysterectomy NEC |
| 7E04512 | TAH - total abdom hysterectomy & bilateral salpingoophorect |
| 7E04900 | TAH - Tot abdom hysterectomy and BSO - bilat salpingophorect |
| 7E04G00 | Total abdominal hysterectomy with conservation of ovaries |
| 7E04B00 | Lapar total abdominal hysterect bilat salpingo-oophorectomy |
| 7E05y00 | Other specified vaginal excision of uterus |
| 7E05z00 | Vaginal excision of uterus NOS |
| 7E05.11 | Schauta radical vaginal hysterectomy |
| 7E05000 | Vaginal hysterocolpectomy and excision of periuterine tissue |
| 7E05200 | Vaginal hysterocolpectomy NEC |
| 7E04y00 | Other specified abdominal excision of uterus |
| 7E04z00 | Abdominal excision of uterus NOS |
| 7E05.00 | Vaginal excision of uterus |
| 7E04.00 | Abdominal excision of uterus |
| 7E04.11 | Abdominal hysterectomy |
| 7E04312 | Hysterectomy NEC |
| 7E04500 | Abdominal hysterectomy and bilateral salpingoophorectomy |
| 7E04511 | Abdominal hysterectomy & bilateral salpingoophorectomy (BSO) |
| 7E04700 | Abdominal hysterectomy and right salpingoopherectomy |
| 7E04711 | Abdominal hysterectomy and left salpingoopherectomy |
| 7E04800 | Abdominal hysterectomy and left salpingoophorectomy |
| 7E04A00 | Abdominal hysterectomy with conservation of ovaries |
| 7E04C00 | Laparoscopic hysterectomy |
| 7E05.12 | Vaginal hysterectomy |
| 7E05300 | Vaginal hysterectomy NEC |
| 7E05400 | Laparoscopic vaginal hysterectomy |
| 7E05500 | Vaginal hysterectomy with conservation of ovaries |
| 7E05y11 | Ward vaginal hysterectomy |
| 7E05600 | Lap assist vag hysterectomy with bilat salpingo-oophorectomy |
| 7E05100 | Vaginal hysterectomy and excision of periuterine tissue NEC |
| 7E04B00 | Lapar total abdominal hysterect bilat salpingo-oophorectomy |
| 7E05600 | Lap assist vag hysterectomy with bilat salpingo-oophorectomy |
| 7E10200 | Bilateral oophorectomy NEC |
| 7E10000 | Bilateral salpingoophorectomy |
| 6127.00 | Partner had tubal ligation |
| ZV25212 | [V]Admission for tubal ligation |
| 7E15.11 | Open bilateral female sterilisation |
| 7E15000 | Open bilateral ligation of fallopian tubes |
| 7E15011 | Pomeroy open bilateral ligation of fallopian tubes |
| 7E15100 | Open bilateral clipping of fallopian tubes |
| 7E15111 | Open bilateral ringing of fallopian tubes |
| 7E15y00 | Other specified open bilateral occlusion of fallopian tubes |
| 7E15z00 | Open bilateral occlusion of fallopian tubes NOS |
| 7E16.00 | Other open occlusion of fallopian tube |
| 7E16.11 | Other open female sterilisation |
| 7E16.12 | Unilateral occlusion of fallopian tube |
| 7E16000 | Open ligation of remaining solitary fallopian tube |
| 7E16100 | Open ligation of fallopian tube NEC |
| 7E16200 | Open clipping of remaining solitary fallopian tube |
| 7E16211 | Open clipping of residual solitary fallopian tube |
| 7E16212 | Open ringing of remaining solitary fallopian tube |
| 7E16300 | Open clipping of fallopian tube NEC |
| 7E16311 | Open ringing of fallopian tube NEC |
| 7E16400 | Open clipping of right fallopian tube |
| 7E16411 | Open ringing of right fallopian tube |
| 7E16500 | Open clipping of left fallopian tube |
| 7E16511 | Open ringing of left fallopian tube |
| 7E16600 | Open ligation of right fallopian tube |
| 7E16700 | Open ligation of left fallopian tube |
| 7E16y00 | Other specified other open occlusion of fallopian tube |
| 7E16z00 | Other open occlusion of fallopian tube NOS |
| 7E17111 | Open removal of ring from fallopian tube NEC |
| 7E18300 | Suture of fallopian tube NEC |
| 7E18400 | Salpingostomy |
| 7E19.00 | Incision of fallopian tube |
| 7E1C.11 | Endoscopic bilateral female sterilisation |
| 7E1C.12 | Laparoscopic bilateral female sterilisation |
| 7E1D.12 | Other endoscopic female sterilisation |
| 7E1D.13 | Other laparoscopic female sterilisation |
| 159A.00 | H/O: tubal ligation |
