# Supplementary material for: Feasibility study to identify women of childbearing age at risk of pregnancy not using any contraception in The Health Improvement Network (THIN) database
Source: BMC Med Inform Decis Mak. 2020 Jul 18;20:164. doi: 10.1186/s12911-020-01184-0 (PMC7368731; doi:10.1186/s12911-020-01184-0)
Supplement: Supplementary file 2 — Additional file 2. Read Code suggestive of infertility and subfertility. List of Read codes. [file 12911_2020_1184_MOESM2_ESM.docx]

# Appendix 2. Read Code suggestive of infertility and subfertility

| **Read code** | **Descriptor** |
| --- | --- |
| 1597.00 | H/O: infertility - female |
| 1597.11 | H/O: female infertility |
| 1AZ2.11 | Infertility problem |
| 3189.11 | Infertility investigation -fem |
| 3189100 | Female infertility test abnormal |
| 8C8..00 | Treatment for infertility |
| 8C82.00 | Female infertility therapy |
| K5B..00 | Infertility - female |
| K5B0.00 | Female infertility of anovulatory origin |
| K5B0000 | Primary anovulatory infertility |
| K5B0100 | Secondary anovulatory infertility |
| K5B0z00 | Female infertility of anovulatory origin NOS |
| K5B1.00 | Female infertility of pituitary - hypothalamic origin |
| K5B1000 | Primary pituitary - hypothalamic infertility |
| K5B1100 | Secondary pituitary - hypothalamic infertility |
| K5B1z00 | Female infertility of pituitary - hypothalamic cause NOS |
| K5B2.00 | Female infertility of tubal origin |
| K5B2000 | Primary tubal infertility |
| K5B2100 | Secondary tubal infertility |
| K5B2z00 | Female infertility of tubal origin NOS |
| K5B3.00 | Female infertility of uterine origin |
| K5B3000 | Primary uterine infertility |
| K5B3100 | Secondary uterine infertility |
| K5B3z00 | Female infertility of uterine origin NOS |
| K5B4.00 | Female infertility of cervical origin |
| K5B4000 | Primary cervical infertility |
| K5B4100 | Secondary cervical infertility |
| K5B4z00 | Female infertility of cervical origin NOS |
| K5B5.00 | Female infertility of vaginal origin |
| K5B5000 | Primary vaginal infertility |
| K5B5100 | Secondary vaginal infertility |
| K5B5z00 | Female infertility of vaginal origin NOS |
| K5B6.00 | Female infertility associated with male factors |
| K5By.00 | Other female infertility |
| K5By000 | Primary infertility unspecified |
| K5By100 | Secondary infertility unspecified |
| K5Byz00 | Other female infertility NOS |
| K5Byz11 | Subfertility |
| K5Bz.00 | Female infertility NOS |
| Kyu9G00 | [X]Female infertility of other origin |
| ZV26.00 | [V]Infertility management |
| ZV26400 | [V]Infertility general advice and counselling |
| ZV26y00 | [V]Other specified infertility management |
| ZV26z00 | [V]Unspecified infertility management |
