# Supplementary material for: Feasibility study to identify women of childbearing age at risk of pregnancy not using any contraception in The Health Improvement Network (THIN) database
Source: BMC Med Inform Decis Mak. 2020 Jul 18;20:164. doi: 10.1186/s12911-020-01184-0 (PMC7368731; doi:10.1186/s12911-020-01184-0)
Supplement: Supplementary file 3 — Additional file 3. Read Code suggestive of menopause. List of Read codes. [file 12911_2020_1184_MOESM3_ESM.docx]

# Appendix 3. Read Code suggestive of menopause

| **Read Code** | **Descriptor** |
| --- | --- |
| 66U..00 | Menopause monitoring |
| 66U..11 | Hormone replacement therapy |
| 66U1.00 | Menopause initial assessment |
| 66U2.00 | Menopause follow-up assessment |
| 66U3.00 | Menopause symptoms present |
| 66U4.00 | Menopause: LH, FSH checked |
| 66U5.00 | Menopause: bone density check |
| 66U6.00 | HRT contraindicated |
| 66U7.00 | HRT started |
| 66U8.00 | HRT side-effects |
| 66U9.00 | HRT changed |
| 66UA.00 | HRT stopped |
| 66UB.00 | HRT: unopposed oestrogen |
| 66UC.00 | HRT: combined oestrog/progest |
| 66UD.00 | Menopause: dietary advice |
| 66UE.00 | Menopause: sexual advice |
| 66UF.00 | Menopause: gen counselling |
| 66UG.00 | Patient refuses HRT |
| 66UH.00 | Hormone replacement therapy bleed pattern - normal |
| 66UI.00 | Hormone replacement therapy bleed pattern - abnormal |
| 66UJ.00 | Hormone replacement therapy bleed pattern - not relevant |
| 66UK.00 | Hormone replacement therapy bleed pattern - no bleeding |
| 66UL.00 | Years on hormone replacement therapy |
| 66UZ.00 | Menopause monitoring NOS |
| K5A..00 | Menopausal and postmenopausal disorders |
| K5A..11 | Postmenopausal disorders |
| K5A0.00 | Premenopausal menorrhagia |
| K5A1.00 | Postmenopausal bleeding |
| K5A2.00 | Menopausal or female climacteric state |
| K5A2000 | Menopausal flushing |
| K5A2011 | Hot flushes - menopausal |
| K5A2100 | Menopausal sleeplessness |
| K5A2200 | Menopausal headache |
| K5A2300 | Menopausal concentration lack |
| K5A2z00 | Menopausal symptoms NOS |
| K5A3.00 | Postmenopausal atrophic vaginitis |
| K5A3.11 | Senile (atrophic) vaginitis |
| K5A4.00 | Artificial menopause state |
| K5Ay.00 | Other menopausal and postmenopausal states |
| K5Az.00 | Menopausal and postmenopausal disorder NOS |
| 1512.00 | Menopause |
| C163111 | Premature menopause NOS |
| K171.00 | Post menopausal atrophic urethritis |
| K171.11 | Post menopausal urethritis |
| Kyu9F00 | [X]Other specified menopausal and perimenopausal disorders |
| N063.11 | Menopausal arthritis |
