# Supplementary material for: Feasibility study to identify women of childbearing age at risk of pregnancy not using any contraception in The Health Improvement Network (THIN) database
Source: BMC Med Inform Decis Mak. 2020 Jul 18;20:164. doi: 10.1186/s12911-020-01184-0 (PMC7368731; doi:10.1186/s12911-020-01184-0)
Supplement: Supplementary file 4 — Additional file 4. Read codes suggestive of long acting reversible contraception (LARC). List of Read codes. [file 12911_2020_1184_MOESM4_ESM.docx]

# Appendix 4. Read codes suggestive of long acting reversible contraception (LARC)

| **Read code** | **Descriptor** |
| --- | --- |
| 61K..00 | Subcutaneous contraceptive |
| 61KA.00 | Insertion of subcutaneous contraceptive |
| 61KB.00 | Check of subcutaneous contraceptive |
| 61KD.00 | Subcutaneous contraceptive in situ |
| 61KE.00 | Subcut contrcptive implnt palp |
| 61KZ.00 | Subcutaneous contraceptive NOS |
| 61B..00 | Depot contraceptive |
| 61B..11 | Depot contraception |
| 61B1.00 | Depot contraceptive given |
| 61B1.11 | Depo-provera injection given |
| 61B2.00 | Depot contraceptive repeated |
| 61B3.00 | Depot contraceptive-no problem |
| 61B4.00 | Depot contraceptive - problem |
| 61BZ.00 | Depot contraceptive NOS |
| 61B6.00 | Depot contraceptive failure |
| 61B6.00 | Depot contraceptive failure |
| 6132.00 | IUD in situ from other agency |
| 615..00 | Intra-uterine contr. device |
| 615..11 | Coil contraception |
| 615..12 | IUD contraception |
| 6151.00 | IUD fitted |
| 6153.00 | IUD re-fitted |
| 6154.00 | IUD checked - no problems |
| 6155.00 | IUD checked - problems |
| 6156.00 | IUD - defaulted from check |
| 6157.00 | IUD fitting awaited |
| 615A.00 | IUD check due |
| 615F.00 | IUD check |
| 615G.00 | IUD in situ |
| 615K.00 | Intrauterine contraceptive device annual review |
| 615L.00 | Intrauterine contraceptive device 6 week check |
| 615M.00 | Intrauterine contraceptive device annual review by telephone |
| 615N.00 | Intrauterine contraceptive device fit by another GP practice |
| 615P.00 | IUCD fitted by other healthcare provider |
| 615R.00 | Intrauterine contracep device checked by other hlth provider |
| 615Z.00 | IUD - NOS |
| 7E09.00 | Intrauterine contraceptive device procedure |
| 7E09.11 | Coil intrauterine contraceptive device procedure |
| 7E09000 | Introduction of intrauterine contraceptive device |
| 7E09011 | Fitting of intrauterine contraceptive device |
| 7E09111 | Change of intrauterine contraceptive device |
| 7E09y00 | Other specified intrauterine contraceptive device |
| 7E09z00 | Intrauterine contraceptive device procedure NOS |
| 962..13 | IUCD contraceptive claim |
| 962..14 | IUD contraceptive claim |
| 962..15 | IUD contraceptive claim |
| 9kA..00 | Intra-uterine contraceptive device fitting - enh serv admin |
| L2D..00 | Retained intrauterine contraceptive device in pregnancy |
| SP03216 | Bleeding due to intrauterine contraceptive device |
| ZV25100 | [V]Intrauterine contraceptive device insertion |
| ZV25112 | [V]Intrauterine contraceptive device insertion |
| ZV25113 | [V]Intrauterine contraceptive device insertion |
| ZV25414 | [V]Intrauterine contraceptive device check |
| ZV25415 | [V]Reinsertion of intrauterine contraceptive device |
| ZV25419 | [V]Intrauterine contraceptive device check |
| ZV2541A | [V]Reinsertion of intrauterine contraceptive device |
| ZV25D00 | [V]Reinsertion of intrauterine contraceptive device |
| ZV25F00 | [V]Intrauterine contraceptive device check |
| ZV45500 | [V]Intrauterine contraceptive device present |
| ZV45511 | [V]Intrauterine contraceptive device present |
| ZV45512 | [V]Intrauterine contraceptive device present |
| SP03217 | Contraception IUCD causing bleeding |
| 9O9..00 | IUD follow-up administration |
| 9O91.00 | IUD check - 1st call |
| 9O92.00 | IUD check - 2nd call |
| 9O93.00 | IUD check - 3rd call |
| 9O94.00 | IUD check - call deleted |
| 9O9Z.00 | IUD follow-up admin. NOS |
| 7E09.12 | Intrauterine device procedure |
| 9O9..11 | Coil follow-up administration |
| ZV25111 | [V]Coil insertion |
| ZV25411 | [V]Coil check |
| ZV25412 | [V]Reinsertion of coil |
| ZV2541C | [V]Coil maintenance |
| ZV25F11 | [V]Coil check |
| ZV45513 | [V]Coil in situ |
| ZV25D11 | [V]Reinsertion of coil |
| 615S.00 | Mirena coil check |
| 7E09400 | Introduction of Mirena coil |
| 61A2.00 | "Morning after" IUD fitted |
| 61A2.11 | Post-coital IUD fitted |
| 61F2.00 | "Morning after" IUCD fitted |
