# Supplementary material for: Feasibility study to identify women of childbearing age at risk of pregnancy not using any contraception in The Health Improvement Network (THIN) database
Source: BMC Med Inform Decis Mak. 2020 Jul 18;20:164. doi: 10.1186/s12911-020-01184-0 (PMC7368731; doi:10.1186/s12911-020-01184-0)
Supplement: Supplementary file 5 — Additional file 5. Read codes suggestive of other ways of contraception. List of Read codes. [file 12911_2020_1184_MOESM5_ESM.docx]

# Appendix 5. Read codes suggestive of other ways of contraception

| **Read Code** | **Descriptor** |
| --- | --- |
| 616..00 | Contraceptive diaphragm |
| 616..11 | CAP contraception |
| 616..12 | Diaphragm contraception |
| 6161.00 | CAP fitted |
| 6162.00 | CAP checked |
| 6163.00 | CAP renewed |
| 6164.00 | CAP - defaulted from check |
| 616Z.00 | CAP - NOS |
| 617..00 | Contraceptive sheath |
| 617..11 | Sheath contraception |
| 617..12 | Spermicide + sheath contracep. |
| 6171.00 | Uses contraceptive sheath |
| 6172.00 | Uses sheath + spermicide |
| 618..00 | Rhythm method contraception |
| 6181.00 | Uses rhythm method |
| 618Z.00 | Rhythm method NOS |
| 61C..00 | Spermicidal contraceptive |
| 61C..11 | Spermicide alone contraception |
| 61D..00 | Contraceptive sponge |
| 61D1.00 | Uses contraceptive sponge |
| 61D2.00 | Uses contr sponge & spermicide |
| 61D4.00 | Contraceptive sponge failure |
| 61DZ.00 | Contraceptive sponge NOS |
| 61E..00 | Sympto-thermal contraception |
| 61E1.00 | Uses sympto-thermal contracepn |
| 61E3.00 | Sympto-thermal c'ceptn-problem |
| 61E4.00 | Symp-thermal c'cep: no problem |
| 61E5.00 | Symto-thermal c'ceptn failure |
| 61EZ.00 | Sympto-thermal contraceptn NOS |
| 61G..00 | Contraception: vasectomy |
| 61H..00 | Contraception: female sterilis |
| 61H1.00 | Female sterilisation failure |
| 61J1.00 | Combined oral contraceptive pill contraindicated |
| 61K..00 | Subcutaneous contraceptive |
| 61KA.00 | Insertion of subcutaneous contraceptive |
| 61KB.00 | Check of subcutaneous contraceptive |
| 61KC.00 | Insert subcutaneous contraceptive implnt othr healthcre prov |
| 61KD.00 | Subcutaneous contraceptive in situ |
| 61KE.00 | Subcut contrcptive implnt palp |
| 61KH.00 | Subcutaneous contraceptive implant not palpable |
| 61KZ.00 | Subcutaneous contraceptive NOS |
| 61N..00 | Transdermal contraceptive |
| 61Q..00 | Partner contraception |
| 61R..00 | Intrauterine system contraception |
| 61T..00 | Uses female condom |
| 61V..00 | Problem with contraception |
| 61V0.00 | Contraceptive sheath problem |
| 61Y..00 | Uses contraception |
| 61Z..00 | Contraception NOS |
| 61b..00 | Uses vaginal hormone releasing ring |
| 8B2L.11 | Condom issued |
| 8B2Y.00 | Female condom issued |
| 9363.00 | Condom scheme card issued |
| 617Z.00 | Contraceptive sheath NOS |
| 8CED.00 | Emergency contraception leaflet given |
| 961..11 | FP1001 - contraception claim |
| 98CA.00 | GMS4 claim - contraception (non IUCD) signed |
| 98CB.00 | GMS4 claim - contraception (non IUCD) sent to HA |
| 98CC.00 | GMS4 claim - contraception (non IUCD) up to date |
| 98CD.00 | GMS4 claim - contraception (non IUCD) due |
| 98CE.00 | GMS4 claim - contraception (non IUCD) due next visit |
| 98CI.00 | GMS4 claim - contraception (non IUCD) paid |
| 98CJ.00 | GMS4 claim - contraception (non IUCD) returned unpaid |
| 98CK.00 | GMS4 claim - contraception (IUCD) signed |
| 98CL.00 | GMS4 claim - contraception (IUCD) sent to HA |
| 98CM.00 | GMS4 claim - contraception (IUCD) due with new IUCD |
| 98CN.00 | GMS4 claim - contraception (IUCD) paid |
| SP03217 | Contraception IUCD causing bleeding |
| 1AB1.00 | Never been sexually active |
| 6122.00 | Not sexually active |
| 685G.00 | No smear - not sexually active |
| 1AB1.11 | Virgin |
