# Supplementary material for: Feasibility study to identify women of childbearing age at risk of pregnancy not using any contraception in The Health Improvement Network (THIN) database
Source: BMC Med Inform Decis Mak. 2020 Jul 18;20:164. doi: 10.1186/s12911-020-01184-0 (PMC7368731; doi:10.1186/s12911-020-01184-0)
Supplement: Supplementary file 6 — Additional file 6. Read codes suggestive of pregnancy. List of Read codes. [file 12911_2020_1184_MOESM6_ESM.docx]

# Appendix 6. Read codes suggestive of pregnancy

| Read codes | Descriptor |
| --- | --- |
| 6166.00 | Pregnant, diaphragm failure |
| 615C.11 | Pregnant, IUD failure |
| 6174.00 | Pregnant, sheath failure |
| lmp |  |
| 1513.00 | Last menstrual period -1st day |
| Delivery |  |
| Ly0..00 | Spontaneous vertex delivery |
| Z257100 | Spontaneous vertex delivery |
| L20..11 | Spontaneous vaginal delivery |
| 63Z..00 | Birth details NOS |
| 63F..00 | Birth details not known |
| ZV27.11 | [V]Live birth |
| 632..00 | Length of labour |
| 6321.00 | 1st stage of labour length |
| 6322.00 | 2nd stage of labour length |
| 6323.00 | 3rd stage of labour length |
| 632Z.00 | Length of labour NOS |
| 6331.00 | Single live birth |
| 63E1.00 | Spontaneous onset of labour |
| 63E2.00 | Normal birth |
| 63E3.00 | Normal labour |
| 7F12.00 | Elective caesarean delivery |
| 7F12000 | Elective upper uterine segment caesarean delivery |
| 7F12100 | Elective lower uterine segment caesarean delivery |
| 7F12111 | Elective lower uterine segment caesarean section (LSCS) |
| 7F12y00 | Other specified elective caesarean delivery |
| 7F12z00 | Elective caesarean delivery NOS |
| 7F13.00 | Other caesarean delivery |
| 7F13000 | Upper uterine segment caesarean delivery NEC |
| 7F13100 | Lower uterine segment caesarean delivery NEC |
| 7F13111 | Lower uterine segment caesarean section (LSCS) NEC |
| 7F13200 | Extraperitoneal caesarean section |
| 7F13300 | Emergency caesarean section |
| 7F13y00 | Other specified other caesarean delivery |
| 7F13z00 | Other caesarean delivery NOS |
| 7F14.00 | Breech extraction delivery |
| 7F14000 | Breech extraction delivery with version |
| 7F14y00 | Other specified breech extraction delivery |
| 7F14z00 | Breech extraction delivery NOS |
| 7F15.00 | Other breech delivery |
| 7F15000 | Spontaneous breech delivery |
| 7F15100 | Assisted breech delivery |
| 7F15y00 | Other specified other breech delivery |
| 7F15z00 | Other breech delivery NOS |
| 7F16.00 | Forceps cephalic delivery |
| 7F16000 | High forceps cephalic delivery with rotation |
| 7F16100 | High forceps cephalic delivery NEC |
| 7F16200 | Mid forceps cephalic delivery with rotation |
| 7F16300 | Mid forceps cephalic delivery NEC |
| 7F16400 | Low forceps cephalic delivery |
| 7F16500 | Trial of forceps delivery |
| 7F16600 | Failed forceps delivery |
| 7F16700 | Barton forceps cephalic delivery with rotation |
| 7F16800 | Dehee forceps cephalic delivery with rotation |
| 7F16900 | Kielland forceps cephalic delivery with rotation |
| 7F16A00 | Scanzoni forceps cephalic delivery with rotation |
| 7F16B00 | Piper forceps delivery |
| 7F16y00 | Other specified forceps cephalic delivery |
| 7F16z00 | Forceps cephalic delivery NOS |
| 7F17.00 | Vacuum delivery |
| 7F17000 | High vacuum delivery |
| 7F17100 | Low vacuum delivery |
| 7F17.11 | Ventouse delivery |
| 7F17200 | Vacuum delivery before full dilation of cervix |
| 7F17300 | Trial of vacuum delivery |
| 7F17y00 | Other specified vacuum delivery |
| 7F17z00 | Vacuum delivery NOS |
| 7F19.00 | Normal delivery |
| 7F19y00 | Other specified normal delivery |
| 7F19z00 | Normal delivery NOS |
| 7F1A000 | Caesarean hysterectomy |
| 7F1B000 | Episiotomy to facilitate delivery |
| 7F1B100 | Symphysiotomy to facilitate delivery |
| 7F1B200 | Pubiotomy to facilitate delivery |
| 7F1B400 | Incision of cervix to facilitate delivery |
| 7F1By00 | Other specified other operation to facilitate delivery |
| 7F1Bz00 | Other operation to facilitate delivery NOS |
| 7F1y.00 | Other specified induction or delivery operations |
| 7F1z.00 | Induction and delivery operations NOS |
| 7F22500 | Normal delivery of placenta |
| L20..00 | Normal delivery in a completely normal case |
| L20..11 | Spontaneous vaginal delivery |
| L200.00 | Normal delivery but ante- or post- natal conditions present |
| L20z.00 | Normal delivery in completely normal case NOS |
| L213100 | Multiple delivery, all by forceps and vacuum extractor |
| L222.11 | Assisted breech delivery |
| L222.12 | Breech delivery |
| L222.13 | Spontaneous breech delivery |
| L290100 | Failed mechanical induction - delivered |
| L296.00 | Vaginal delivery following previous caesarean section |
| L300300 | Obstructed labour due to breech presentation |
| L308.00 | Failed forceps unspecified |
| L308000 | Other failed forceps, unspecified |
| L308200 | Other failed forceps with antenatal problem |
| L308z00 | Failed forceps NOS |
| L309.00 | Failed ventouse extraction unspecified |
| L309000 | Other failed ventouse extraction, unspecified |
| L309200 | Other failed ventouse extraction with antenatal problem |
| L309z00 | Failed ventouse extraction NOS |
| L395.00 | Forceps delivery |
| L395000 | Forceps delivery unspecified |
| L395100 | Forceps delivery - delivered |
| L395.11 | Keilland's forceps delivery |
| L395.12 | Neville - Barnes forceps delivery |
| L395.13 | Simpson's forceps delivery |
| L395200 | Low forceps delivery |
| L395300 | Mid-cavity forceps delivery |
| L395400 | Delivery by combination of forceps and vacuum extractor |
| L395500 | Mid-cavity forceps with rotation |
| L395z00 | Forceps delivery NOS |
| L396.00 | Vacuum extractor delivery |
| L396000 | Vacuum extractor delivery unspecified |
| L396100 | Vacuum extractor delivery - delivered |
| L396.11 | Ventouse delivery |
| L396z00 | Vacuum extractor delivery NOS |
| L397.00 | Breech extraction |
| L397000 | Breech extraction unspecified |
| L397100 | Breech extraction - delivered |
| L397z00 | Breech extraction NOS |
| L398.00 | Caesarean delivery |
| L398000 | Caesarean delivery unspecified |
| L398100 | Caesarean delivery - delivered |
| L398200 | Caesarean section - pregnancy at term |
| L398300 | Delivery by elective caesarean section |
| L398400 | Delivery by emergency caesarean section |
| L398500 | Delivery by caesarean hysterectomy |
| L398600 | Caesarean delivery following previous Caesarean delivery |
| L398z00 | Caesarean delivery NOS |
| L441.00 | Caesarean wound disruption |
| L441000 | Caesarean wound disruption unspecified |
| L441100 | Caesarean wound disruption - delivered with p/n complication |
| L441200 | Caesarean wound disruption with postnatal complication |
| L441z00 | Caesarean wound disruption NOS |
| Ly0..00 | Spontaneous vertex delivery |
| Ly0..00 | Spontaneous vertex delivery |
| Ly1..00 | Spontaneous breech delivery |
| Lyu5100 | [X]Other and unspecified forceps delivery |
| Lyu5200 | [X]Other single delivery by caesarean section |
| Lyu5300 | [X]Other assisted breech delivery |
| Z243500 | Normal length of first stage of labour |
| Z243700 | Normal first stage of labour |
| Z246100 | Duration of labour |
| Z246111 | Length of labour |
| Z248.00 | Normal labour |
| Z253300 | Normal rate of delivery |
| Z254200 | Delivered by low forceps delivery |
| Z254300 | Delivered by mid-cavity forceps delivery |
| Z254500 | Delivered by caesarean section - pregnancy at term |
| Z254800 | Deliveries by spontaneous breech delivery |
| Z255600 | Normal length of second stage of labour |
| Z255900 | Normal second stage of labour |
| Z256100 | Normal length of third stage of labour |
| Z257.00 | Delivery normal |
| Z257100 | Spontaneous vertex delivery |
| Z257.12 | Spontaneous vaginal delivery |
| Z257.13 | SVD - Spontaneous vaginal delivery |
| Z257.14 | FTND - Full term normal delivery |
| Z257.15 | ND - Normal delivery |
| Z258.00 | Delivery problem |
| ZV27000 | [V]Single live birth |
| ZV27.11 | [V]Live birth |
| 6331.00 | Single live birth |
| 63...00 | Birth details |
| ZV27z00 | [V]Unspecified delivery outcome |
| ZV27y00 | [V]Other specified outcome of delivery |
| ZV27.00 | [V]Outcome of delivery |
| 633..00 | Outcome of delivery |
| 633Z.00 | Outcome of delivery NOS |
| L0...00 | Pregnancy with abortive outcome |
| L0y..00 | Other specified pregnancy with abortive outcome |
| L0z..00 | Pregnancy with abortive outcome NOS |
| Lyu0.00 | [X]Pregnancy with abortive outcome |
| 7E08200 | Evacuation of products of conception from uterus NEC |
| 7E06011 | Hysterotomy & evacuation retained products conception NEC |
| L04..11 | Miscarriage |
| L02..11 | Missed miscarriage |
| L04..00 | Spontaneous abortion |
| L05..12 | Termination of pregnancy |
| 7E08600 | Termination of pregnancy NEC |
| 7E08400 | Suction termination of pregnancy |
| 7E06011 | Hysterotomy & evacuation retained products conception NEC |
| 7E07112 | Curette of retained products of conception from uterus NEC |
| 7E08100 | Dilation cervix & evacuation products conception uterus NEC |
| ZV27.11 | [V]Live birth |
| L21y100 | Other multiple pregnancy - delivered |
| L21z100 | Multiple pregnancy NOS - delivered |
| L213.00 | Multiple delivery |
| ZV27000 | [V]Single live birth |
| 633a.00 | Birth of child |
| 633..13 | Triplet birth |
| 633..14 | Twin birth |
| 634..11 | Delivery - sex of baby |
| L210100 | Twin pregnancy - delivered |
| L211100 | Triplet pregnancy - delivered |
| L212100 | Quadruplet pregnancy - delivered |
| L213000 | Multiple delivery, all spontaneous |
| L213200 | Multiple delivery, all by caesarean section |
| L294100 | Grand multiparity - delivered |
| L295100 | Elderly primigravida - delivered |
| 6331.00 | Single live birth |
| 6333.00 | Twins - both live born |
| 6334.00 | Twins - 1 still + 1 live born |
| 6335.00 | Twins - both still born |
| 6344.00 | 2 female babies |
| 6345.00 | 1 male + 1 female baby |
| 6346.00 | 3 male babies |
| 6347.00 | 2 male + 1 female babies |
| 6348.00 | 1 male + 2 female babies |
| 6349.00 | 3 female babies |
| 7F19100 | Water birth delivery |
| 6341.00 | Baby male |
| 6342.00 | Baby female |
| 62P..00 | Infant feeding method |
| 64...14 | Infant feeding method |
| 62PZ.00 | Infant feeding method NOS |
| 635..00 | Maturity of baby |
| 635..11 | Full term baby |
| 7F1..00 | Induction and delivery operations |
| 7F10.00 | Surgical induction of labour |
| 7F10y00 | Other specified surgical induction of labour |
| 7F10z00 | Surgical induction of labour NOS |
| 7F11.00 | Other induction of labour |
| 7F11000 | Oxytocic induction of labour |
| 7F11100 | Induction of labour using prostaglandins |
| 7F11200 | Syntocinon induction of labour |
| 7F11300 | Medical induction of labour |
| 7F11y00 | Other specified other induction of labour |
| 7F11z00 | Other induction of labour NOS |
| 7L16000 | Intravenous induction of labour |
| L290.11 | Failed mechanical induction of labour |
| L291100 | Failed medical or unspecified induction - delivered |
| L291.11 | Failed medical induction of labour |
| Lyu4000 | [X]Other failed induction of labour |
| Lyu6A00 | [X]Infection of caesarean section wound following delivery |
| Q032.00 | Fetus or neonate affected by forceps delivery |
| Q033.00 | Fetus or neonate affected by vacuum extraction delivery |
| ZVu2A00 | [X]Other multiple births, all liveborn |
| ZVu2B00 | [X]Other multiple births, some liveborn |
| ZVu2C00 | [X]Other multiple births, all stillborn |
| L3...00 | Complications occurring during labour and delivery |
| L3y..00 | Other specified complications of labour or delivery |
| L3z..00 | Complications of labour and delivery NOS |
| ZV3..00 | [V]Healthy liveborn infants according to type of birth |
| ZV3y.00 | [V]Other multiple birth, unspecified |
| ZV3yz00 | [V]Other multiple birth, unspecified, NOS |
| ZV3z.00 | [V]Unspecified birth |
| ZV3zz00 | [V]Unspecified birth, NOS |
| Lyu4.00 | [X]Complications of labour and delivery |
| Lyu4A00 | [X]Labour+delivery complicated by other cord complications |
| Lyu4E00 | [X]Oth pulmonary complicatns/anaesthesia during lab+delivery |
| Lyu4F00 | [X]Oth complicatn/spinl+epidur anaesths during lab+delivery |
| Lyu4G00 | [X]Other complications of anaesthesia during labour+delivery |
| Lyu4H00 | [X]Other infection during labour |
| Lyu4K00 | [X]Other specified complications of labour and delivery |
| Lyu4L00 | [X]Obstructed labour due to fetopelv disproportion, unspec |
| Lyu4N00 | [X]Labour & delivery complicated by fetal stress, unspecif |
| Lyu4P00 | [X]Complication of anaesthesia during labour and deliv unsp |
| Lyu5.00 | [X]Delivery |
| Z1H4.00 | Pain relief in labour |
| L14..11 | Premature labour |
| Q21..00 | Intrauterine hypoxia and birth asphyxia |
| Z23AG00 | Hypertonic lower uterine segment during labour |
| Z23G.00 | Uterine observation in labour |
| Z24..00 | Labour observations |
| Z25..00 | Delivery observations |
| ZV27y00 | [V]Other specified outcome of delivery |
| ZV27z00 | [V]Unspecified delivery outcome |
| L29..00 | Other problems affecting labour |
| L29y.00 | Other problems affecting labour |
| L29yz00 | Other problems affecting labour NOS |
| L29z.00 | Problems affecting labour NOS |
| L29zz00 | Problems affecting labour NOS |
| L30..00 | Obstructed labour |
| L30A.00 | Obstructed labour due to unusually large fetus |
| L30y.00 | Other causes of obstructed labour |
| L30yz00 | Other causes of obstructed labour NOS |
| L30z.00 | Obstructed labour NOS |
| L30zz00 | Obstructed labour NOS |
| ZV3y000 | [V]Other multiple birth, unspecified, born in hospital |
| ZV3z000 | [V]Unspecified birth, born in hospital |
| L31..00 | Abnormal forces of labour |
| L31z.00 | Abnormality of forces of labour NOS |
| L31zz00 | Abnormality of forces of labour NOS |
| ZV3y100 | [V]Other multiple birth, unspecified, born before hospital |
| ZV3z100 | [V]Unspecified birth, born before admission to hospital |
| L32..00 | Long labour |
| L32z.00 | Prolonged labour NOS |
| ZV3y200 | [V]Other multiple birth, unspecified, not hospitalised |
| ZV3z200 | [V]Unspecified birth, not hospitalised |
| ZV34.00 | [V]Other multiple birth, mates live born |
| ZV34z00 | [V]Other multiple birth, mates live born, NOS |
| L34y.00 | Other vulval and perineal trauma during delivery |
| L34yz00 | Other vulval/perineal trauma during delivery NOS |
| L34z.00 | Vulval/perineal trauma during delivery NOS |
| L34zz00 | Vulval/perineal trauma during delivery NOS |
| L34..12 | Vulval delivery trauma |
| ZV35.00 | [V]Other multiple birth, mates stillborn |
| ZV35z00 | [V]Other multiple birth, mates stillborn, NOS |
| L35z.00 | Obstetric trauma NOS |
| L35zz00 | Obstetric trauma NOS |
| Q036.00 | Fetus or neonate affected by precipitate delivery |
| ZV36.00 | [V]Other multiple birth, mates live and stillborn |
| ZV36z00 | [V]Other multiple birth, mates live and stillborn NOS |
| L38..00 | Complications of anaesthesia during labour and delivery |
| L38A.00 | Failed or difficult intubation during labour and delivery |
| L38X.00 | Complication of anaesthesia during labour and deliv unsp |
| L39..00 | Other complications of labour and delivery NEC |
| L39y.00 | Other complications of labour and delivery |
| L39yz00 | Other complications of labour and delivery NOS |
| L39z.00 | Complications of labour and delivery NOS |
| L39zz00 | Complications of labour and delivery NOS |
| Lyu4200 | [X]Other abnormalities of forces of labour |
| L43y.00 | Other obstetric pulmonary embolism |
| Lyu4300 | [X]Obstructed labour due/other malposition+malpresentation |
| Lyu4400 | [X]Obstructd labour due to oth maternal pelvic abnormalities |
| Lyu4500 | [X]Obstructed labour due to other abnormalities of fetus |
| Lyu4600 | [X]Other specified obstructed labour |
| Lyu4800 | [X]Labour+delivery complicat/oth evidence of fetal distress |
| Lyu4900 | [X]Labour+delivery complicated by other cord entanglement |
| Lyu5000 | [X]Other single spontaneous delivery |
| Lyu5400 | [X]Other manipulation-assisted delivery |
| Lyu5500 | [X]Other specified assisted single delivery |
| Lyu5600 | [X]Other multiple delivery |
| Lyu5700 | [X]Assisted single delivery, unspecified |
| Lyu5800 | [X]Multiple delivery, unspecified |
| 7F1A.00 | Other methods of delivery |
| 7F1Ay00 | Other specified other method of delivery |
| 7F1Az00 | Other method of delivery NOS |
| 7F1B.00 | Other operations to facilitate delivery |
| L142.00 | Early onset of delivery |
| L142z00 | Early onset of delivery NOS |
| L142.11 | Premature delivery |
| L18z100 | Medical condition NOS during pregnancy - baby delivered |
| Q208.00 | Cerebral oedema due to birth injury |
| Z213.00 | Care of mother in labour |
| Z23B400 | Contraction of uterus during labour |
| ZV24000 | [V]Examination immediately after delivery |
| Z241.00 | Labour established |
| Z243.00 | Observation of first stage of labour |
| Z244.00 | Observation of pattern of labour |
| Z245.00 | Observation of blood loss in labour |
| Z246.00 | Observation of measures of labour |
| Z246A00 | Total duration of labour |
| Z247.00 | Device-associated observation of labour |
| Z249.00 | Labour problem |
| Z253.00 | Observation of speed of delivery |
| Z253.11 | Speed of delivery |
| Z253.12 | Rate of delivery |
| Z254.00 | Observation of pattern of delivery |
| Z254A00 | Abnormal delivery |
| Z254B00 | Brow delivery |
| Z254C00 | Face delivery |
| Z254E00 | Multiple birth |
| Z254E11 | Multiple birth delivery |
| Z255.00 | Observation of second stage of labour |
| Z255A00 | Observation of delivery push in labour |
| Z256.00 | Observation of third stage of labour |
| ZV27500 | [V]Other multiple birth, all live born |
| ZV27600 | [V]Other multiple birth, some live born |
| ZV27700 | [V]Other multiple birth, all stillborn |
| L29y000 | Other problems affecting labour unspecified |
| L29z000 | Problems affecting labour NOS unspecified |
| L29y100 | Other problems affecting labour - delivered |
| L29z100 | Problems affecting labour NOS - delivered |
| L292.00 | Maternal pyrexia during labour, unspecified |
| L292z00 | Unspecified maternal pyrexia during labour NOS |
| L29y200 | Other problems affecting labour with antenatal problem |
| L29z200 | Problems affecting labour NOS with antenatal problem |
| L293.00 | Septicaemia during labour |
| L293z00 | Septicaemia during labour NOS |
| L300.00 | Obstructed labour due to fetal malposition |
| L300z00 | Obstructed labour due to fetal malposition NOS |
| L30y000 | Other causes of obstructed labour unspecified |
| L30z000 | Obstructed labour NOS, unspecified |
| L301.00 | Obstructed labour caused by bony pelvis |
| L301z00 | Obstructed labour caused by bony pelvis NOS |
| L30y100 | Other causes of obstructed labour - delivered |
| L30z100 | Obstructed labour NOS - delivered |
| L302.00 | Obstructed labour caused by pelvic soft tissues |
| L302z00 | Obstructed labour caused by pelvic soft tissues NOS |
| L30y200 | Other causes of obstructed labour with antenatal problem |
| L30z200 | Obstructed labour NOS with antenatal problem |
| L307.00 | Failed trial of labour unspecified |
| L307z00 | Failed trial of labour NOS |
| L31z000 | Abnormality of forces of labour NOS unspecified |
| L31z100 | Abnormality of forces of labour NOS - delivered |
| L31z200 | Abnormality of forces of labour NOS with antenatal problem |
| L313.00 | Precipitate labour |
| L313z00 | Precipitate labour NOS |
| L321.00 | Prolonged labour unspecified |
| L321z00 | Prolonged labour NOS |
| L323.00 | Delayed delivery of second twin, triplet etc |
| L323z00 | Delayed delivery second twin etc NOS |
| L340.00 | First degree perineal tear during delivery |
| L340z00 | First degree perineal tear during delivery NOS |
| ZV34000 | [V]Other multiple birth, born in hospital, mates live born |
| L34y000 | Other vulval/perineal trauma during delivery, unspecified |
| L34z000 | Vulval/perineal trauma during delivery NOS unspec |
| L341.00 | Second degree perineal tear during delivery |
| L341z00 | Second degree perineal tear during delivery NOS |
| ZV34100 | [V]Other multiple birth, born before hospital, mates live |
| L34y100 | Other vulval/perineal trauma during delivery- delivered |
| L34z100 | Vulval/perineal trauma during delivery NOS - delivered |
| L342.00 | Third degree perineal tear during delivery |
| L342z00 | Third degree perineal tear during delivery NOS |
| ZV34200 | [V]Other multiple birth, not hospitalised, mates live born |
| L34y200 | Other vulval/perineal trauma during delivery + p/n problem |
| L34z200 | Vulval/perineal trauma during delivery NOS with p/n problem |
| L343.00 | Fourth degree perineal tear during delivery |
| L343z00 | Fourth degree perineal tear during delivery NOS |
| L344.00 | Unspecified perineal laceration during delivery |
| L344z00 | Unspecified perineal laceration during delivery NOS |
| L345.00 | Vulval and perineal haematoma during delivery |
| L345z00 | Vulval and perineal haematoma during delivery NOS |
| L345.12 | Vulval and perineal haematoma during delivery |
| ZV35000 | [V]Other multiple birth, born in hospital, mates stillborn |
| ZV35100 | [V]Other multiple birth,born before hospital,mates stillborn |
| ZV35200 | [V]Other multiple birth, not hospitalised, mates stillborn |
| ZV36000 | [V]Other multiple birth, born in hospital, mates live+still |
| ZV36100 | [V]Other multiple birth, before hospital, mates live+still |
| ZV36200 | [V]Other multiple birth, not hospitalised, mates live+still |
| L386.00 | Toxic reaction to local anaesthesia during labour and deliv |
| L387.00 | Spinal/epidural anesth-induced headache dur labour/delivery |
| L388.00 | Cardiac comps of anaesthesia during labour and delivery |
| L389.00 | CNS comps of anaesthesia during labour and delivery |
| L39y000 | Other complications of labour and delivery unspecified |
| L39z000 | Complications of labour and delivery NOS, unspecified |
| L39y100 | Other complications of labour and delivery - delivered |
| L39z100 | Complications of labour and delivery NOS - delivered |
| L39y200 | Other complications of labour and delivery - deliv +p/n prob |
| L39z200 | Complications of labour and delivery NOS - del + p/n problem |
| L39y300 | Other complications of labour and delivery with a/n problem |
| L39z300 | Complications of labour and delivery NOS with antenatal prob |
| L39y400 | Other complications of labour and delivery with p/n problem |
| L39z400 | Complications of labour and delivery NOS with p/n problem |
| Q432.00 | Preterm delivery associated jaundice |
| H472.00 | Asp pneumonitis due to anaesthesia during labour and deliv |
| 7F1B000 | Episiotomy to facilitate delivery |
| 7F1A100 | Destructive operation to facilitate delivery |
| 7F1A200 | Cleidotomy of fetus to facilitate delivery |
| 7F1A300 | Drainage of hydrocephalus of fetus to facilitate delivery |
| 7F1A400 | Trial of labour NEC |
| 7F25.13 | Monitoring during labour |
| L126600 | Eclampsia in labour |
| L142100 | Early onset of delivery - delivered |
| L166600 | Urinary tract infection following delivery |
| Z239600 | Segments of uterus distinguishable in labour |
| Z239700 | State of upper segment retraction during labour |
| L240100 | Congenital abnormality of uterus - baby delivered |
| L240111 | Bicornuate uterus - baby delivered |
| Z243100 | First stage of labour established |
| Z243200 | First stage of labour not established |
| Z243300 | Progress of labour - first stage |
| Z243400 | Rapid first stage of labour |
| Z243411 | Rapid progress in first stage of labour |
| Z243600 | Slow progress in first stage of labour |
| Z243800 | First stage of labour problem |
| L244100 | Other uterine/pelvic floor abnormality - baby delivered |
| Z244100 | Observation of duration of labour |
| L244111 | Cystocele - baby delivered |
| L244112 | Rectocele - baby delivered |
| L244200 | Other uterine/pelvic floor abn - delivered+postpartum compl |
| Z244200 | Long duration of labour |
| L244211 | Cystocele - delivered with postpartum complication |
| L244212 | Rectocele - delivered with postpartum complication |
| Z244300 | Short duration of labour |
| Z244400 | Late onset of labour |
| Z244411 | Postmature labour |
| Z244500 | Relation of onset of labour to due date |
| L246100 | Other cervical abnormality - baby delivered |
| L246200 | Other cervical abnormality - baby delivered+postpartum compl |
| Z246200 | Onset of labour first stage |
| L246211 | Polyp of cervix - baby delivered+postpartum complication |
| Z246211 | Start of labour |
| L246212 | Stenosis of cervix - baby delivered+postpartum complication |
| Z246311 | Onset of labour pains |
| Z246700 | Onset of second stage of labour |
| Z246900 | Duration of second stage of labour |
| L247100 | Vaginal abnormality - baby delivered |
| L247111 | Septate vagina - baby delivered |
| L247112 | Stenosis of vagina - baby delivered |
| Z253100 | Slow rate of delivery |
| Z253200 | Rapid rate of delivery |
| Z253211 | Precipitate delivery |
| Z254900 | Vaginal delivery |
| Z255100 | Second stage of labour established |
| Z255200 | Second stage of labour not established |
| Z255300 | Progess of second stage of labour |
| Z255311 | Progess of delivery |
| Z255400 | Rapid second stage of labour |
| Z255700 | Failure to progress in second stage of labour |
| Z255711 | No progress with delivery |
| Z255712 | No progress in second stage of labour |
| Z255800 | Second stage of labour problem |
| Z256200 | Prolonged third stage of labour |
| Z256300 | Speed of delivery of placenta |
| Z256311 | Rate of delivery of placenta |
| Z262600 | Complete placenta at delivery |
| Z262700 | Incomplete placenta at delivery |
| Z262711 | Incomplete delivery of placenta |
| L263300 | Labour and delivery complicated by fetal heart rate anomaly |
| Z263300 | Condition of membranes at delivery |
| L263400 | Labour and delivery complic by meconium in amniotic fluid |
| L263500 | Lab+del comp fetal ht rate anom wth meconium in amnio fluid |
| L263600 | Labour+delivery complicatd by biochem evidence/fetal stress |
| Z265900 | Umbilical cord not around baby's neck at delivery |
| L292000 | Unspecified maternal pyrexia during labour, unspecified |
| L292100 | Unspecified maternal pyrexia during labour - delivered |
| L292200 | Unspecified maternal pyrexia during labour with a/n problem |
| L293000 | Septicaemia during labour unspecified |
| L293100 | Septicaemia during labour - delivered |
| L293200 | Septicaemia during labour with antenatal problem |
| L300000 | Obstructed labour due to fetal malposition unspecified |
| L300100 | Obstructed labour due to fetal malposition - delivered |
| L300200 | Obstructed labour due to fetal malposition with a/n problem |
| L300400 | Obstructed labour due to face presentation |
| L300500 | Obstructed labour due to brow presentation |
| L300600 | Obstructed labour due to shoulder presentation |
| L300700 | Obstructed labour due to compound presentation |
| L301000 | Obstructed labour caused by bony pelvis unspecified |
| L301100 | Obstructed labour caused by bony pelvis - delivered |
| L301200 | Obstructed labour caused by bony pelvis with a/n problem |
| L301300 | Obstructed labour due to deformed pelvis |
| L301400 | Obstructed labour due to generally contracted pelvis |
| L301500 | Obstructed labour due to pelvic inlet contraction |
| L301600 | Obstruct labour due pelvic outlet and mid-cavity contract |
| L301700 | Obstructed labour due abnormality of maternal pelv organs |
| L302000 | Obstructed labour caused by pelvic soft tissues unspecified |
| L302100 | Obstructed labour caused by pelvic soft tissues - delivered |
| L302200 | Obstructed labour caused by pelvic soft tissues + a/n prob |
| L307000 | Other failed trial of labour unspecified |
| L307100 | Other failed trial of labour - delivered |
| L307200 | Other failed trial of labour with antenatal problem |
| L313000 | Precipitate labour unspecified |
| L313100 | Precipitate labour - delivered |
| L313200 | Precipitate labour with antenatal problem |
| L321000 | Unspecified prolonged labour, unspecified |
| L321100 | Unspecified prolonged labour - delivered |
| L321200 | Unspecified prolonged labour with antenatal problem |
| L323000 | Delayed delivery second twin unspecified |
| L323100 | Delayed delivery second twin - delivered |
| L340000 | First degree perineal tear during delivery, unspecified |
| L340100 | First degree perineal tear during delivery - delivered |
| L340200 | First degree perineal tear during delivery with p/n problem |
| L340300 | Labial tear during delivery |
| L340400 | Fourchette tear during delivery |
| L340500 | Vulval tear during delivery |
| L340600 | Vaginal tear during delivery |
| L341000 | Second degree perineal tear during delivery, unspecified |
| L341100 | Second degree perineal tear during delivery - delivered |
| L341200 | Second degree perineal tear during delivery with p/n prob |
| L342000 | Third degree perineal tear during delivery, unspecified |
| L342100 | Third degree perineal tear during delivery - delivered |
| L342200 | Third degree perineal tear during delivery with p/n problem |
| L343000 | Fourth degree perineal tear during delivery, unspecified |
| L343100 | Fourth degree perineal tear during delivery - delivered |
| L343200 | Fourth degree perineal tear during delivery with p/n problem |
| L344000 | Unspecified perineal laceration during delivery, unspecified |
| L344100 | Unspecified perineal laceration during delivery - delivered |
| L344200 | Unspecified perineal laceration during delivery + p/n prob |
| L345000 | Vulval and perineal haematoma during delivery, unspecified |
| L345100 | Vulval and perineal haematoma during delivery - delivered |
| L345200 | Vulval and perineal haematoma during delivery + p/n problem |
| L351000 | Rupture of uterus during and after labour unspecified |
| L351100 | Rupture of uterus during and after labour - delivered |
| L351200 | Rupture of uterus during/after labour - deliv with p/n prob |
| L351300 | Rupture of uterus during/after labour with postnatal problem |
| L355100 | Other obstetric pelvic organ damage - delivered |
| 7F19000 | Manually assisted vaginal delivery |
| 7M35000 | Gas and air analgesia in labour |
| 7M35011 | Entonox analgesia in labour |
| 956..00 | HSA1-therap. abort. green form |
| L05..00 | Legally induced abortion |
| L05..11 | Elective abortion |
| L05..13 | Therapeutic abortion |
| L050.00 | Legal abortion unspecified |
| L050000 | Unspecified legal abortion + genital tract/pelvic infection |
| L050100 | Unspecified legal abortion + delayed/excessive haemorrhage |
| L050200 | Unspecified legal abortion + damage to pelvic organs/tissues |
| L050300 | Unspecified legal abortion with renal failure |
| L050400 | Unspecified legal abortion with metabolic disorder |
| L050500 | Unspecified legal abortion with shock |
| L050600 | Unspecified legal abortion with embolism |
| L050w00 | Unspecified legal abortion with other specified complication |
| L050x00 | Unspecified legal abortion with complication NOS |
| L050y00 | Unspecified legal abortion with no mention of complication |
| L050z00 | Unspecified legal abortion NOS |
| L052.00 | Legal abortion complete |
| L052.11 | Medical abortion - complete |
| L052.12 | Surgical abortion - complete |
| L052000 | Complete legal abortion + genital tract or pelvic infection |
| L052100 | Complete legal abortion with delayed/excessive haemorrhage |
| L052200 | Complete legal abortion + damage to pelvic organs or tissues |
| L052300 | Complete legal abortion with renal failure |
| L052400 | Complete legal abortion with metabolic disorder |
| L052500 | Complete legal abortion with shock |
| L052600 | Complete legal abortion with embolism |
| L052w00 | Complete legal abortion with other specified complication |
| L052x00 | Complete legal abortion with complication NOS |
| L052y00 | Complete legal abortion with no mention of complication |
| L052z00 | Complete legal abortion NOS |
| L06..00 | Illegally induced abortion |
| L06..11 | Criminal abortion |
| L06..12 | Self-induced abortion |
| L060.00 | Illegal abortion unspecified |
| L060000 | Unspec illegal abortion + genital tract or pelvic infection |
| L060100 | Unspec illegal abortion + delayed or excessive haemorrhage |
| L060200 | Unspecified illegal abortion + pelvic organ/tissue damage |
| L060300 | Unspecified illegal abortion with renal failure |
| L060400 | Unspecified illegal abortion with metabolic disorder |
| L060500 | Unspecified illegal abortion with shock |
| L060600 | Unspecified illegal abortion with embolism |
| L060w00 | Unspecified illegal abortion + other specified complication |
| L060x00 | Unspecified illegal abortion with complication NOS |
| L060y00 | Unspecified illegal abortion with no mention of complication |
| L060z00 | Unspecified illegal abortion NOS |
| L062.00 | Illegal abortion complete |
| L062000 | Complete illegal abortion + genital tract/pelvic infection |
| L062100 | Complete illegal abortion + delayed or excessive haemorrhage |
| L062200 | Complete illegal abortion + pelvic organ/tissue damage |
| L062300 | Complete illegal abortion with renal failure |
| L062400 | Complete illegal abortion with metabolic disorder |
| L062500 | Complete illegal abortion with shock |
| L062600 | Complete illegal abortion with embolism |
| L062w00 | Complete illegal abortion with other specified complication |
| L062x00 | Complete illegal abortion with complication NOS |
| L062y00 | Complete illegal abortion with no mention of complication |
| L062z00 | Complete illegal abortion NOS |
| L06z.00 | Illegally induced abortion NOS |
| L08..00 | Failed attempted abortion |
| L08w.00 | Failed attempted abortion with other specified complication |
| L08x.00 | Failed attempted abortion with complication NOS |
| L08y.00 | Failed attempted abortion with no mention of complication |
| L08z.00 | Failed attempted abortion NOS |
| L097100 | Readmission for retained produc of concept, legal abortion |
| L097200 | Readmission for retained produc of concept, illegal abortion |
| 7E08411 | Vacuum termination of pregnancy |
| 7E08500 | Dilation of cervix and extraction termination of pregnancy |
| 7E06600 | Hysterotomy and termination of pregnancy |
| 7E07011 | Dilation cervix uteri & curettage for termination pregnancy |
| 7E07113 | Curettage of uterus for termination of pregnancy NEC |
| 7E07114 | Curettage of uterus for termination of pregnancy NEC |
| ZV25311 | [V]Admission for termination of pregnancy (TOP) |
| ZV25313 | [V]Admission for termination of pregnancy |
| Q486.00 | Fetal death due to termination of pregnancy |
| L0...00 | Pregnancy with abortive outcome |
| L02..00 | Missed abortion |
| L02..12 | Silent miscarriage |
| L04..00 | Spontaneous abortion |
| L04z.00 | Spontaneous abortion NOS |
| L10zz11 | Inevitable abortion |
| L040.00 | Spontaneous abortion unspecified |
| L040w00 | Unspec spontaneous abortion + other specified complication |
| L040x00 | Unspecified spontaneous abortion with complication NOS |
| L040y00 | Unspec spontaneous abortion without mention of complication |
| L040z00 | Unspecified spontaneous abortion NOS |
| L041z11 | Retained products after spontaneous abortion |
| L042.00 | Spontaneous abortion complete |
| L042w00 | Complete spontaneous abortion + other specified complication |
| L042x00 | Complete spontaneous abortion with complication NOS |
| L042y00 | Complete spontaneous abortion + no mention of complication |
| L042z00 | Complete spontaneous abortion NOS |
| L043.00 | Inevitable abortion unspecified |
| L043x00 | Unspecified inevitable abortion with unspec complication |
| L043y00 | Unspecified inevitable abortion with OS complication |
| L043z00 | Unspecified inevitable abortion without complication |
| L043.11 | Inevitable miscarriage unspecified |
| L043x11 | Unspecified inevitable miscarriage with unspec complication |
| L043y11 | Unspecified inevitable miscarriage with OS complication |
| L043z11 | Unspecified inevitable miscarriage without complication |
| L045.00 | Inevitable abortion complete |
| L045x00 | Complete inevitable abortion with unspecified complication |
| L045y00 | Complete inevitable abortion with OS complication |
| L045z00 | Complete inevitable abortion without complication |
| L045.11 | Inevitable miscarriage complete |
| L045x11 | Complete inevitable miscarriage with unspecified comp |
| L045y11 | Complete inevitable miscarriage with OS complication |
| L045z11 | Complete inevitable miscarriage without complication |
| L040000 | Unspec spontaneous abortion + genital tract/pelvic infection |
| L040011 | Spontaneous abortion with sepsis |
| L040100 | Unspec spontaneous abortion + delayed/excessive haemorrhage |
| L040111 | Spontaneous abortion with heavy bleeding |
| L040200 | Unspec spontaneous abortion + pelvic organ/tissue damage |
| L040300 | Unspecified spontaneous abortion with renal failure |
| L040400 | Unspecified spontaneous abortion with metabolic disorder |
| L040500 | Unspecified spontaneous abortion with shock |
| L040600 | Unspecified spontaneous abortion with embolism |
| L040900 | Inevitable miscarriage |
| L042000 | Complete spontaneous abortion + genital tract/pelvic infect |
| L042100 | Complete spontaneous abortion +delayed/excessive haemorrhage |
| L042200 | Complete spontaneous abortion + pelvic organ/tissue damage |
| L042300 | Complete spontaneous abortion with renal failure |
| L042400 | Complete spontaneous abortion with metabolic disorder |
| L042500 | Complete spontaneous abortion with shock |
| L042600 | Complete spontaneous abortion with embolism |
| L043000 | Unspec inev abor comp by genital tract and pelvic infect |
| L043011 | Unspec inev miscarriage comp by genital tract pelvic infec |
| L043100 | Unspec inevit abortion comp by delayed or excessive haemorr |
| L043111 | Unsp inevitable mis comp by delayed or excessive haemorrhage |
| L043200 | Unspecified inevitable abortion complicated by embolism |
| L043211 | Unspecified inevitable miscarriage complicated by embolism |
| L045000 | Complete inev abor comp by genital tract and pelvic infec |
| L045011 | Complete inev misc compl by genital tract and pelvic infec |
| L045100 | Complete inevitable abor comp by delayed or excessive haem |
| L045111 | Complete inevitable miscar comp by delayed or excessive haem |
| L045200 | Complete inevitable abortion complicated by embolism |
| L045211 | Complete inevitable miscarriage complicated by embolism |
| L097000 | Readmis for retain products of concept, spontaneous abortion |
| L100100 | Threatened abortion - delivered |
| 7E08800 | Dilation and curettage removal of missed abortion |
| L0y..00 | Other specified pregnancy with abortive outcome |
| L0z..00 | Pregnancy with abortive outcome NOS |
| Lyu0.00 | [X]Pregnancy with abortive outcome |
| Lyu0200 | [X]Other abortion |
| L07..00 | Unspecified abortion |
| L07z.00 | Unspecified abortion NOS |
| L09..00 | Complications following abortion/ectopic/molar pregnancies |
| L09y.00 | Other specified complication following abortive pregnancy |
| L09yz00 | Other specified complication NOS follow abortive pregnancy |
| L09z.00 | Complication NOS following abortion/ectopic/molar pregnancy |
| L09..11 | Complications following abortion/ectopic/molar pregnancies |
| L070.00 | Unspecified abortion |
| L070w00 | Unspecified abortion with other specified complication |
| L070x00 | Unspecified abortion with complication NOS |
| L070y00 | Unspecified abortion with no mention of complication |
| L070z00 | Unspecified abortion NOS |
| L072.00 | Unspecified abortion complete |
| L072w00 | Unspecified complete abortion + other specified complication |
| L072x00 | Unspecified complete abortion with complication NOS |
| L072y00 | Unspecified complete abortion + no mention of complication |
| L072z00 | Unspecified complete abortion NOS |
| L090.00 | Genital or pelvic infection following abortive pregnancy |
| L090y00 | Sepsis NOS following abortion/ectopic/molar pregnancy |
| L090z00 | Septicaemia NOS following abortive pregnancy |
| L09y000 | Acute liver necrosis following abortive pregnancy |
| L091.00 | Delayed/excessive haemorrhage following abortive pregnancy |
| L091z00 | Delayed/excess haemorrhage NOS following abortive pregnancy |
| L09y100 | Cardiac arrest following abortive pregnancy |
| L092.00 | Pelvic organ or tissue damage following abortive pregnancy |
| L092z00 | Pelvic organ or tissue damage NOS follow abortive pregnancy |
| L09y200 | Cardiac failure following abortive pregnancy |
| L093.00 | Renal failure following abortive pregnancy |
| L093z00 | Renal failure NOS following abortive pregnancy |
| L09y300 | Cerebral anoxia following abortive pregnancy |
| L094.00 | Metabolic disorder following abortive pregnancy |
| L09y400 | Urinary tract infection following abortive pregnancy |
| L095.00 | Shock following abortive pregnancy |
| L096.00 | Embolism following abortive pregnancy |
| L096z00 | Embolism NOS following abortive pregnancy |
| L096.11 | Embolus following abortive pregnancy |
| L097.00 | Readmission for abortive pregnancy (NHS codes) |
| L097.11 | Readmission for retained products of conception (NHS codes) |
| Z22B900 | Continuing pregnancy after abortion of sibling fetus |
| L070000 | Unspecified abortion with genital tract or pelvic infection |
| L070100 | Unspecified abortion with delayed or excessive haemorrhage |
| L070200 | Unspecified abortion with damage to pelvic organs or tissues |
| L070300 | Unspecified abortion with renal failure |
| L070400 | Unspecified abortion with metabolic disorder |
| L070500 | Unspecified abortion with shock |
| L070600 | Unspecified abortion with embolism |
| 7E08y00 | Other specified evacuation of contents of uterus |
| 7F20.00 | Instrument removal retained products conception deliv uterus |
| L072000 | Unspecified complete abortion + genital tract/pelvic infect |
| 7F21.00 | Manual removal retained products conception delivered uterus |
| L072100 | Unspecified complete abortion +delayed/excessive haemorrhage |
| L072200 | Unspecified complete abortion + pelvic organ/tissue damage |
| L072300 | Unspecified complete abortion with renal failure |
| L072400 | Unspecified complete abortion with metabolic disorder |
| L072500 | Unspecified complete abortion with shock |
| L072600 | Unspecified complete abortion with embolism |
| L090000 | Endometritis following abortive pregnancy |
| L090100 | Parametritis following abortive pregnancy |
| L090200 | Pelvic peritonitis following abortive pregnancy |
| L090300 | Salpingitis following abortive pregnancy |
| L090400 | Salpingo-oophoritis following abortive pregnancy |
| L091000 | Afibrinogenaemia following abortive pregnancy |
| L091100 | Defibrination syndrome following abortive pregnancy |
| L091200 | Intravascular haemolysis following abortive pregnancy |
| L092000 | Bladder damage following abortive pregnancy |
| L092100 | Bowel damage following abortive pregnancy |
| L092200 | Broad ligament damage following abortive pregnancy |
| L092300 | Cervix damage following abortive pregnancy |
| L092400 | Periurethral tissue damage following abortive pregnancy |
| L092500 | Uterus damage following abortive pregnancy |
| L092600 | Vaginal damage following abortive pregnancy |
| L093000 | Oliguria following abortive pregnancy |
| L093100 | Acute renal failure following abortive pregnancy |
| L093200 | Renal shutdown following abortive pregnancy |
| L093300 | Renal tubular necrosis following abortive pregnancy |
| L093400 | Uraemia following abortive pregnancy |
| L096000 | Air embolism following abortive pregnancy |
| L096100 | Amniotic fluid embolism following abortive pregnancy |
| L096200 | Blood-clot embolism following abortive pregnancy |
| L096300 | Fat embolism following abortive pregnancy |
| L096400 | Pulmonary embolism following abortive pregnancy |
| L096500 | Pyaemic embolism following abortive pregnancy |
| L096600 | Septic embolism following abortive pregnancy |
| L096700 | Soap embolism following abortive pregnancy |
| L097300 | Readmission for retained produc of concept, unspec abortion |
| 6232.00 | A/N care: recurrent aborter |
| DELIVERY | |
| 6341.00 | Baby male |
| 6342.00 | Baby female |
| 62P..00 | Infant feeding method |
| 64...14 | Infant feeding method |
| 62PZ.00 | Infant feeding method NOS |
| 635..00 | Maturity of baby |
| 635..11 | Full term baby |
| 635..12 | Postmature baby |
| 635..13 | Premature baby |
| 6351.00 | Baby premature 36-38 weeks |
| 6352.00 | Baby v. premature 32-36 weeks |
| 6353.00 | Baby extremely prem.28-32 week |
| 6354.00 | Baby full term maturity |
| 6355.00 | Baby post-mature |
| 6356.00 | Baby premature 26-28 weeks |
| 6357.00 | Baby premature 24-26 weeks |
| 6358.00 | Baby premature 39 weeks |
| 6359.00 | Baby premature 38 weeks |
| 635A.00 | Baby premature 37 weeks |
| 635B.00 | Baby premature 36 weeks |
| 635Z.00 | Baby maturity NOS |
| 637Z.00 | Birth head circumference NOS |
| L266100 | Large-for-dates - delivered |
| Q11..11 | Baby born premature |
| Q110.00 | Very premature - less than 1000g or less than 28 weeks |
| Q110.11 | Immature baby |
| Q111.00 | Premature - weight 1000g-2499g or gestation of 28-37weeks |
| Q112.00 | Extreme immaturity |
| Q112.11 | Extreme prematurity - less than 28 weeks |
| Q113.00 | Light for gestational age |
| Q114.00 | Low birthweight |
| Q114000 | Birth weight 1000-2499 g |
| Q115.00 | Extremely low birth weight infant |
| Q115000 | Birth weight 999 g or less |
| Q11z.00 | Born premature NOS |
| Q12..00 | Disorders relating to long gestation and high birthweight |
| Q12..11 | Large baby born |
| Q120.00 | Very large baby - weight greater than 4500gm |
| Q122.00 | Postmature infant - greater than 42 weeks gestation, unspec |
| Q12z.00 | Large or postmature infant NOS |
| Lyu6B00 | [X]Vaginitis following delivery |
| Lyu6C00 | [X]Cervicitis following delivery |
| 8CH..00 | Post partum care |
| L18z.00 | Medical condition NOS in pregnancy/childbirth/puerperium |
| ZV24.00 | [V]Postpartum care and examination |
| ZV24y00 | [V]Other specified postpartum care and examination |
| ZV24z00 | [V]Unspecified postpartum care and examination |
| ZV24.11 | [V]Postnatal care and examination |
| Lyu6000 | [X]Other infection of genital tract following delivery |
| Lyu6100 | [X]Other genitourinary tract infections following delivery |
| L18z000 | Medical condition NOS - unsp whether in pregnancy/puerperium |
| Z242.00 | Labour not established |
| Z255C00 | No desire to push in labour |
| Z255D00 | Ability to push in labour |
| Z255E00 | Pushing effectively in labour |
| Z255F00 | Not pushing well in labour |
| Z255G00 | Urge to push in labour |
| Z255H00 | Reluctant to push in labour |
| Z255I00 | Pushing voluntarily in labour |
| Z255J00 | Pushing involuntarily in labour |
| Z255B11 | Wants to push in labour |
| Z255D11 | Observation of ability to push in labour |
| Z255E11 | Pushing well in labour |
| Z286.00 | Pelvis adequate for delivery |
| Z287.00 | Pelvis not adequate for delivery |
| Z288.00 | Problem of pelvis for delivery |
| L351.00 | Rupture of uterus during and after labour |
| L351z00 | Rupture of uterus during and after labour NOS |
| L393.00 | Acute renal failure following labour and delivery |
| L393z00 | Post-delivery acute renal failure NOS |
| L39y500 | Maternal exhaustion |
| 633A.00 | Live birth surviving more than one year |
| L142000 | Early onset of delivery unspecified |
| L240200 | Cong abnormality uterus - baby delivered + postpartum compl |
| L240211 | Bicornuate uterus - baby delivered + postpartum complication |
| L241000 | Tumour of uterine body affecting obstetric care |
| L241011 | Uterine fibroid affecting obstetric care |
| L241100 | Tumour of uterine body - baby delivered |
| L247200 | Vaginal abnormality - baby delivered+postpartum complication |
| L247211 | Septate vagina - baby delivered with postpartum complication |
| L247212 | Stenosis of vagina - baby delivered+postpartum complication |
| L248100 | Vulval abnormality - baby delivered |
| L248111 | Persistent hymen - baby delivered |
| L248112 | Rigid perineum - baby delivered |
| L263311 | Maternal care for fetal hypoxia |
| L281300 | Prem rupture of membranes onset of labour within 24 hours |
| L281500 | Prem rupture of membranes onset of labour after 24 hours |
| L355000 | Other obstetric pelvic organ damage unspecified |
| L393000 | Post-delivery acute renal failure unspecified |
| L393100 | Post-delivery acute renal failure - delivered with p/n prob |
| L393200 | Post-delivery acute renal failure with postnatal problem |
| Desde -91 dias (relativo) |  |
| Hasta 0 dias (relativo) |  |
|  |  |
| Conjunto de events: ECTOPIC |  |
| 584E.00 | Antenatal ultrasound confirms ectopic pregnancy |
| 7E13100 | Excision of ectopic ovarian pregnancy |
| 7E13300 | Excision of ruptured ectopic tubal pregnancy |
| 7E19000 | Removal of products of conception from fallopian tube |
| 7E19011 | Removal of ectopic pregnancy from fallopian tube |
| L03..00 | Ectopic pregnancy |
| L030.00 | Abdominal pregnancy |
| L031.00 | Tubal pregnancy |
| L031000 | Fallopian tube pregnancy |
| L031100 | Gravid fallopian tube rupture |
| L031200 | Tubal abortion |
| L031z00 | Tubal pregnancy NOS |
| L032.00 | Ovarian pregnancy |
| L03y.00 | Other ectopic pregnancy |
| L03y000 | Cervical pregnancy |
| L03y100 | Cornual pregnancy |
| L03y200 | Membranous pregnancy |
| L03y300 | Combined or heterotopic pregnancy |
| L03y400 | Mural pregnancy |
| L03y500 | Intraligamentous pregnancy |
| L03y600 | Mesenteric pregnancy |
| L03y700 | Angular pregnancy |
| L03y800 | Mesometric pregnancy |
| L03yz00 | Other ectopic pregnancy NOS |
| L03z.00 | Ectopic pregnancy NOS |
| Lyu0000 | [X]Other ectopic pregnancy |
| L03y100 | Cornual pregnancy |
| L03y500 | Intraligamentous pregnancy |
| L03y700 | Angular pregnancy |
| L032.00 | Ovarian pregnancy |
| OTHER CODES PREGNANCY | |
| 584D.00 | Antenatal ultrasound confirms intra-uterine pregnancy |
| 13H7.00 | Unwanted pregnancy |
| 13H8.00 | Illegitimate pregnancy |
| 13Hd.00 | Teenage pregnancy |
| 13S..00 | Pregnancy benefits |
| 13SZ.00 | Pregnancy benefit NOS |
| 4453.00 | Serum pregnancy test positive |
| 4654.00 | Urine pregnancy test positive |
| 6166.00 | Pregnant, diaphragm failure |
| 6174.00 | Pregnant, sheath failure |
| 62...00 | Patient pregnant |
| 62...13 | Pregnancy care |
| 621..00 | Patient currently pregnant |
| 621..11 | Pregnancy confirmed |
| 6211.00 | Pregnant - urine test confirms |
| 6212.00 | Pregnant - blood test confirms |
| 6213.00 | Pregnant - V.E. confirms |
| 6215.00 | Pregnant - on abdom. palpation |
| 6216.00 | Pregnant - planned |
| 6217.00 | Pregnant - unplanned - wanted |
| 6218.00 | Pregnant -unplanned-not wanted |
| 621C.00 | Unplanned pregnancy |
| 621Z.00 | Patient pregnant NOS |
| 62H3.00 | Rh screen - 1st preg. sample |
| 62H4.00 | Rh screen - 2nd preg. sample |
| 62H5.00 | Rh screen - 3rd preg. sample |
| 62O7.00 | Pregnancy prolonged - 41 weeks |
| 62O8.00 | Pregnancy prolonged - 42 weeks |
| 7F2B100 | Ultrasound monitoring of early pregnancy |
| L15..11 | Post-term pregnancy |
| L150.00 | Post-term pregnancy |
| L150z00 | Post-term pregnancy NOS |
| L163300 | Pregnancy care of habitual aborter |
| Z212.11 | Pregnancy care |
| Z227.00 | Confirmation of pregnancy |
| Z22A900 | Unwanted pregnancy |
| Z22AA00 | Wanted pregnancy |
| Z22AB00 | Unplanned pregnancy |
| Z22AB11 | Accidental pregnancy |
| Z22AD00 | Presentation of pregnancy |
| Z22AD11 | Reported conception - pregnancy |
| Z22C311 | Pregnancy duration |
| Z231.00 | Gravid uterus present |
| ZV22.00 | [V]Normal pregnancy |
| ZV22200 | [V]Pregnancy confirmed |
| ZV22300 | [V]Pregnant state, incidental |
| ZV61900 | [V]Other unwanted pregnancy |
| Z22AC00 | Pregnancy with uncertain dates |
| Z22C500 | Estimated date of conception |
| Z22C511 | EDC - Estimated date of conception |
| Z22C100 | Estimated date of delivery from last period |
| Z22C200 | Estimated date of delivery from last normal period |
| 1514.11 | Due to deliver - EDC |
| 1514.12 | Estimated date of delivery |
| 2684.00 | O/E - VE - gravid uterus |
| 271..00 | O/E - gravid uterus size |
| 271Z.00 | O/E - gravid uterus size NOS |
| 44Cy.00 | Serum pregnancy associated plasma protein-A MoM measurement |
| 44Cz.00 | Plasma pregnancy associated plasma protein-A MoM |
| 62a..00 | Pregnancy review |
| 62a..11 | Review of pregnancy |
| 62O..12 | Static weight gain pregnancy |
| 67A2.00 | Diet in pregnancy advice |
| 67A3.00 | Pregnancy smoking advice |
| 67A4.00 | Pregnancy exercise advice |
| 67A5.00 | Pregnancy alcohol advice |
| 67A6.00 | Drugs in pregnancy advice |
| 67A7.00 | Pregnancy dental advice |
| 67AB.00 | Preg. prescription exempt adv. |
| 67AE.00 | Folic acid advice in first trimester of pregnancy |
| 67AZ.00 | Pregnancy advice NOS |
| 7F0..00 | Fetus and gravid uterus operations |
| 7F0..12 | Fetus & gravid uterus ops |
| 7F06.00 | Operations on gravid uterus |
| 7F06000 | Cerclage of cervix of gravid uterus |
| 7F06012 | Shirodkar suture in pregnancy |
| 7F06100 | Removal of cerclage from cervix of gravid uterus |
| 7F06200 | Repositioning of retroverted gravid uterus |
| 7F06y00 | Other specified operation on gravid uterus |
| 7F06z00 | Operation on gravid uterus NOS |
| 7F0y.00 | Other specified operations on fetus or gravid uterus |
| 7F0z.00 | Fetus and gravid uterus operations NOS |
| 7N61100 | [SO]Gravid uterus |
| 8B68.00 | Pregnancy prophylactic therapy |
| 8B7..11 | Pregnancy vitamin/iron prophyl |
| 8B74.00 | Iron supplement in pregnancy |
| 8B75.00 | Vitamin supplement - pregnancy |
| 9Ea0.00 | Risk life pregnant woman greater than if pregnancy terminatd |
| L126500 | Eclampsia in pregnancy |
| L12B.00 | Proteinuric hypertension of pregnancy |
| L12z.00 | Unspecified hypertension in pregnancy/childbirth/puerperium |
| L130.00 | Mild hyperemesis gravidarum |
| L130000 | Mild hyperemesis unspecified |
| L130200 | Mild hyperemesis-not delivered |
| L130z00 | Mild hyperemesis gravidarum NOS |
| L131.00 | Hyperemesis gravidarum with metabolic disturbance |
| L131000 | Hyperemesis gravidarum with metabolic disturbance unsp |
| L131200 | Hyperemesis gravidarum with metabolic disturbance - not del |
| L131z00 | Hyperemesis gravidarum with metabolic disturbance NOS |
| L132.00 | Late vomiting of pregnancy |
| L132000 | Late pregnancy vomiting unspecified |
| L132100 | Late pregnancy vomiting - delivered |
| L132200 | Late pregnancy vomiting - not delivered |
| L132z00 | Late pregnancy vomiting NOS |
| L13y000 | Other pregnancy vomiting unspecified |
| L13y100 | Other pregnancy vomiting - delivered |
| L13y200 | Other pregnancy vomiting - not delivered |
| L13z000 | Unspecified pregnancy vomiting unspecified |
| L13z100 | Unspecified pregnancy vomiting - delivered |
| L13z200 | Unspecified pregnancy vomiting - not delivered |
| L15..00 | Prolonged or post-term pregnancy |
| L150000 | Post-term pregnancy unspecified |
| L150100 | Post-term pregnancy - delivered |
| L150200 | Post-term pregnancy - not delivered |
| L15z.00 | Prolonged pregnancy NOS |
| L16..00 | Other pregnancy complication NEC |
| L161.00 | Oedema or excessive weight gain in pregnancy no hypertension |
| L161000 | Oedema or excessive weight gain in pregnancy, unspecified |
| L161100 | Oedema or excessive weight gain in pregnancy, delivered |
| L161.11 | Excessive weight gain in pregnancy |
| L161.13 | Gestational oedema |
| L161300 | Oedema or excessive weight gain in pregnancy - not delivered |
| L161z00 | Oedema or excessive weight gain in pregnancy NOS |
| L162100 | Unspecified renal disease in pregnancy - delivered |
| L162200 | Unspecified renal disease in pregnancy - del with p/n comp |
| L16D.00 | Excessive weight gain in pregnancy |
| L16E.00 | Pregnancy pruritus |
| L16y500 | Abdominal pain in pregnancy |
| L2B..00 | Low weight gain in pregnancy |
| Lyu3000 | [X]Other multiple gestation |
| Lyu3100 | [X]Other complications specific to multiple gestation |
| Lyu3400 | [X]Maternal care for other abnormalities of gravid uterus |
| M240500 | Alopecia of pregnancy |
| Z22..00 | Pregnancy observations |
| Z225.00 | Normal pregnancy |
| Z226.00 | Pregnancy problem |
| Z229.00 | Observation of position of pregnancy |
| Z229100 | Intrauterine pregnancy |
| Z22A.00 | Observation of pattern of pregnancy |
| Z22A100 | Low risk pregnancy |
| Z22A200 | High risk pregnancy |
| Z22A211 | HRP - High risk pregnancy |
| Z22A400 | Early stage of pregnancy |
| Z22A500 | Biochemical pregnancy |
| Z22A600 | Teenage pregnancy |
| Z22A700 | Surrogate pregnancy |
| Z22AC00 | Pregnancy with uncertain dates |
| Z22B.00 | Observation of quantity of pregnancy |
| Z22B900 | Continuing pregnancy after abortion of sibling fetus |
| Z22C.00 | Observation of measures of pregnancy |
| Z22C313 | Duration of pregnancy |
| Z22D.00 | Observation of viability of pregnancy |
| Z22D100 | Viable pregnancy |
| Z22D200 | Non-viable pregnancy |
| Z22D311 | Query viability of pregnancy |
| Z23..00 | Observation of gravid uterus |
| Z234.00 | Observation of size of gravid uterus |
| Z234100 | Gravid uterus large-for-dates |
| Z234200 | Gravid uterus small-for-dates |
| Z234300 | Observation of height of gravid uterus |
| Z235.00 | Observation of shape of pregnant abdomen |
| Z235100 | Ovoid pregnant abdomen |
| Z235200 | Rounded pregnant abdomen |
| Z235211 | Globular pregnant abdomen |
| Z235300 | Transversely enlarged pregnant abdomen |
| Z235400 | Pendulous pregnant abdomen |
| Z236.00 | Observation of arrangement of gravid uterus |
| Z236100 | Normal position of gravid uterus |
| Z236200 | Gravid uterus central |
| Z236300 | Pregnant uterus displaced laterally |
| Z236400 | Gravid uterus deviated to left |
| Z236500 | Gravid uterus deviated to right |
| Z237.00 | Observation of sensation of gravid uterus |
| Z237100 | Non-tender scar of gravid uterus |
| Z237200 | Tender scar of gravid uterus |
| Z23D.00 | Observation of measures of gravid uterus |
| Z23D100 | Girth of pregnant abdomen |
| Z23D200 | Pregnant abdomen observation |
| Z23E.00 | Gravid uterus normal |
| Z23F.00 | Gravid uterus problem |
| Z6S1.11 | Gravity assisted positioning |
| ZV13900 | [V]PH comp of pregnancy, childbirth and the puerperium |
| ZV22100 | [V]Other normal pregnancy supervision |
| ZV22.11 | [V]Supervision of normal pregnancy |
| ZV22400 | [V]Supervision of other normal pregnancy |
| ZV22y00 | [V]Other specified pregnant state |
| ZV22z00 | [V]Unspecified pregnant state |
| ZV23.00 | [V]High-risk pregnancy supervision |
| ZV23000 | [V]Pregnancy with history of infertility |
| ZV23100 | [V]Pregnancy with history of trophoblastic disease |
| ZV23111 | [V]Pregnancy with history of hydatidiform mole |
| ZV23112 | [V]Pregnancy with history of vesicular mole |
| ZV23200 | [V]Pregnancy with history of abortion |
| ZV23400 | [V]Pregnancy with other poor obstetric history |
| ZV23500 | [V]Pregnancy with other poor reproductive history |
| ZV23600 | [V]Supervisn/pregnancy wth history insufficnt antenatal care |
| ZV23800 | [V]Supervision of high-risk pregnancy due to social problems |
| ZV23y00 | [V]Other specified high-risk pregnancy |
| ZV23z00 | [V]Unspecified high-risk pregnancy |
| ZV4J000 | [V]Problems related to unwanted pregnancy |
| ZVu2300 | [X]Supervision of other normal pregnancy |
| ZVu2400 | [X]Supervision of preg with oth poor reprod obstet history |
| ZVu2500 | [X]Supervision of other high-risk pregnancies |
| L13..00 | Excessive pregnancy vomiting |
| L13..11 | Hyperemesis gravidarum |
| L13..12 | Hyperemesis of pregnancy |
| L13y.00 | Other pregnancy vomiting |
| L13yz00 | Other pregnancy vomiting NOS |
| L13z.00 | Unspecified pregnancy vomiting |
| L13zz00 | Unspecified pregnancy vomiting NOS |
| L191.00 | Continuing pregnancy after abortion of one fetus or more |
| L142000 | Early onset of delivery unspecified |
| Z22CF00 | Date symptom of pregnancy first noted |
| Z22C300 | Length of gestation |
| Z23B100 | Date false contractions first detected |
| Z244500 | Relation of onset of labour to due date |
| 63CE.00 | One of twins |
| L21..00 | Multiple pregnancy |
| L21..11 | Gestation - multiple |
| L210.00 | Twin pregnancy |
| L210000 | Twin pregnancy unspecified |
| L210200 | Twin pregnancy with antenatal problem |
| L210z00 | Twin pregnancy NOS |
| L211.00 | Triplet pregnancy |
| L211000 | Triplet pregnancy unspecified |
| L211200 | Triplet pregnancy with antenatal problem |
| L211z00 | Triplet pregnancy NOS |
| L212.00 | Quadruplet pregnancy |
| L212000 | Quadruplet pregnancy unspecified |
| L212200 | Quadruplet pregnancy with antenatal problem |
| L212z00 | Quadruplet pregnancy NOS |
| L21y.00 | Other multiple pregnancy |
| L21y000 | Other multiple pregnancy unspecified |
| L21y200 | Other multiple pregnancy with antenatal problem |
| L21yz00 | Other multiple pregnancy NOS |
| L21z.00 | Multiple pregnancy NOS |
| L21z000 | Multiple pregnancy NOS, unspecified |
| L21z200 | Multiple pregnancy NOS with antenatal problem |
| L21zz00 | Multiple pregnancy NOS |
| L228.00 | Multiple pregnancy with malpresentation |
| L228000 | Multiple pregnancy with malpresentation unspecified |
| L228200 | Multiple pregnancy with malpresentation with antenatal prob |
| L228z00 | Multiple pregnancy with malpresentation NOS |
| Z22B100 | Single pregnancy |
| Z22B500 | Quintuplet pregnancy |
| Z22B600 | Sextuplet pregnancy |
| Z22B700 | Septulet pregnancy |
| ZV22000 | [V]First normal pregnancy supervision |
| ZV23700 | [V]Supervision of very young primigravida |
| Z221.12 | FIRST TIME MOTHER |
| 6776.00 | Preg. termination counselling |
| 67A..00 | Pregnancy advice |
| 67A7.11 | Care of teeth advice -in preg. |
| E201C00 | Phantom pregnancy |
| E202E00 | Fear of pregnancy |
| L032.00 | Ovarian pregnancy |
| L130.11 | Morning sickness |
| L161.12 | Maternal obesity syndrome |
| Z2...00 | Pregnancy, childbirth and puerperium observations |
| Z21..00 | Care relating to reproduction and pregnancy |
| Z22A300 | Concealed pregnancy |
| Z22A800 | Undiagnosed pregnancy |
| Z22BA00 | Contin pregnancy after intrauterine death of sibling fetus |
| Z22C314 | Weeks pregnant |
| Z22CF00 | Date symptom of pregnancy first noted |
| Z22D300 | Uncertain viability of pregnancy |
| ZG9..00 | Advice relating to pregnancy and fertility |
| ZV72400 | [V]Pregnancy examination or test, pregnancy unconfirmed |
| ZV72900 | [V]Pregnancy examination and test |
| 8HHf.00 | Refer to early pregnancy unit |
| Lyu6B00 | [X]Vaginitis following delivery |
| Lyu6C00 | [X]Cervicitis following delivery |
| L18z.00 | Medical condition NOS in pregnancy/childbirth/puerperium |
| Lyu6000 | [X]Other infection of genital tract following delivery |
| Lyu6100 | [X]Other genitourinary tract infections following delivery |
| L18z000 | Medical condition NOS - unsp whether in pregnancy/puerperium |
| Z242.00 | Labour not established |
| Z255C00 | No desire to push in labour |
| Z255D00 | Ability to push in labour |
| Z255E00 | Pushing effectively in labour |
| Z255F00 | Not pushing well in labour |
| Z255G00 | Urge to push in labour |
| Z255H00 | Reluctant to push in labour |
| Z255I00 | Pushing voluntarily in labour |
| Z255J00 | Pushing involuntarily in labour |
| Z255B11 | Wants to push in labour |
| Z255D11 | Observation of ability to push in labour |
| Z255E11 | Pushing well in labour |
| Z286.00 | Pelvis adequate for delivery |
| Z287.00 | Pelvis not adequate for delivery |
| Z288.00 | Problem of pelvis for delivery |
| L351.00 | Rupture of uterus during and after labour |
| L351z00 | Rupture of uterus during and after labour NOS |
| L393.00 | Acute renal failure following labour and delivery |
| L393z00 | Post-delivery acute renal failure NOS |
| L39y500 | Maternal exhaustion |
| 633A.00 | Live birth surviving more than one year |
| L142000 | Early onset of delivery unspecified |
| L240200 | Cong abnormality uterus - baby delivered + postpartum compl |
| L240211 | Bicornuate uterus - baby delivered + postpartum complication |
| L241000 | Tumour of uterine body affecting obstetric care |
| L241011 | Uterine fibroid affecting obstetric care |
| L241100 | Tumour of uterine body - baby delivered |
| L247200 | Vaginal abnormality - baby delivered+postpartum complication |
| L247211 | Septate vagina - baby delivered with postpartum complication |
| L247212 | Stenosis of vagina - baby delivered+postpartum complication |
| L248100 | Vulval abnormality - baby delivered |
| L248111 | Persistent hymen - baby delivered |
| L248112 | Rigid perineum - baby delivered |
| L263311 | Maternal care for fetal hypoxia |
| L281300 | Prem rupture of membranes onset of labour within 24 hours |
| L281500 | Prem rupture of membranes onset of labour after 24 hours |
| L355000 | Other obstetric pelvic organ damage unspecified |
| L393000 | Post-delivery acute renal failure unspecified |
| L393100 | Post-delivery acute renal failure - delivered with p/n prob |
| L393200 | Post-delivery acute renal failure with postnatal problem |
| 63E..00 | Labour details |
| 7F1..11 | Labour operations |
| L140.00 | Threatened premature labour |
| L140z00 | Threatened premature labour NOS |
| L141.00 | Other threatened labour |
| L141z00 | Other threatened labour NOS |
| L030000 | Delivery of viable fetus in abdominal pregnancy |
| L35z000 | Obstetric trauma NOS, unspecified |
| L35y000 | Other obstetric trauma unspecified |
| L35z100 | Obstetric trauma NOS - delivered |
| L35y100 | Other obstetric trauma - delivered |
| 62V0.00 | Home delivery planned |
| 62C1.00 | Short stay delivery booking |
| 62B2.00 | Home delivery booked |
| 62C2.00 | Full stay delivery booking |
| 62B3.00 | G.P. unit delivery booking |
| 62B5.00 | Private home delivery booking |
| 62B6.00 | Delivery booking place changed |
| 62B7.00 | Domino delivery |
| 62B8.00 | Midwife unit delivery booking |
| L140000 | Threatened premature labour unspecified |
| L140100 | Threatened premature labour - not delivered |
| L141000 | Other threatened labour unspecified |
| L141100 | Other threatened labour - not delivered |
| Z212100 | Delivery place planned |
| Z212200 | Home delivery planned |
| Z212300 | Delivery place booked |
| L244000 | Other uterine/pelvic floor abnormal affecting obstetric care |
| L244011 | Cystocele affecting obstetric care |
| L244012 | Rectocele affecting obstetric care |
| L247000 | Vaginal abnormality affecting obstetric care |
| L247011 | Septate vagina affecting obstetric care |
| L247012 | Stenosis of vagina affecting obstetric care |
| L248000 | Vulval abnormality affecting obstetric care |
| L248011 | Persistent hymen affecting obstetric care |
| L248012 | Rigid perineum affecting obstetric care |
| 62B1.00 | Delivery: no place booked |
| 622..00 | Antenatal care: gravida No. |
| 6221.00 | Antenatal care: primigravida |
| 6222.00 | Antenatal care: 2nd pregnancy |
| 6223.00 | Antenatal care: 3rd pregnancy |
| 6224.00 | Antenatal care: multip |
| 622Z.00 | Antenatal care: gravida NOS |
| 6241.00 | A/N care: elderly primip. |
| 6282.00 | A/N care:10yrs+since last preg |
| 6283.00 | A/N care: primip. < 17 years |
| 6284.00 | A/N care: primip. > 30 years |
