# Supplementary material for: Feasibility study to identify women of childbearing age at risk of pregnancy not using any contraception in The Health Improvement Network (THIN) database
Source: BMC Med Inform Decis Mak. 2020 Jul 18;20:164. doi: 10.1186/s12911-020-01184-0 (PMC7368731; doi:10.1186/s12911-020-01184-0)
Supplement: Supplementary file 7 — Additional file 7. Read codes suggestive of vasectomy. List of Read codes. [file 12911_2020_1184_MOESM7_ESM.docx]

# Appendix 7. Read codes suggestive of vasectomy

| **Read Code** | **Descriptor** |
| --- | --- |
| 14E4.00 | H/O: vasectomy |
| 49N..00 | Semen analysis, post vasectomy |
| 6124.00 | Partner had vasectomy |
| 61G..00 | Contraception: vasectomy |
| 7C11000 | Bilateral vasectomy for contraception |
| 7C11012 | Vasectomy NEC |
| 7C11100 | Unilateral vasectomy for contraception |
| 8M10.00 | Reversal vasectomy requested |
| 9Ngd.00 | Post vasectomy special clearance to stop contraception given |
| K27y600 | Vasectomy scar |
| ZV25213 | [V]Admission for vasectomy |
| 6124.11 | Partner sterilised |
| 14E2.00 | H/O: infertility - male |
| 14E2.11 | H/O: male infertility |
| 8C83.00 | Male infertility therapy |
| K26..00 | Male infertility |
| K26y.00 | Infertility due to extratesticular cause |
| K26y000 | Infertility due to drug therapy |
| K26y100 | Infertility due to infective cause |
| K26y200 | Infertility due to efferent duct obstruction |
| K26y300 | Infertility due to radiation |
| K26y400 | Infertility in systemic disease |
| K26yz00 | Infertility due to extratesticular cause NOS |
| K26z.00 | Male infertility NOS |
