# Supplementary figures and images for: Feasibility study to identify women of childbearing age at risk of pregnancy not using any contraception in The Health Improvement Network (THIN) database
Source: BMC Med Inform Decis Mak. 2020 Jul 18;20:164. doi: 10.1186/s12911-020-01184-0 (PMC7368731; doi:10.1186/s12911-020-01184-0)

**Appendix 8. Questionnaire sent to the PCP**

**
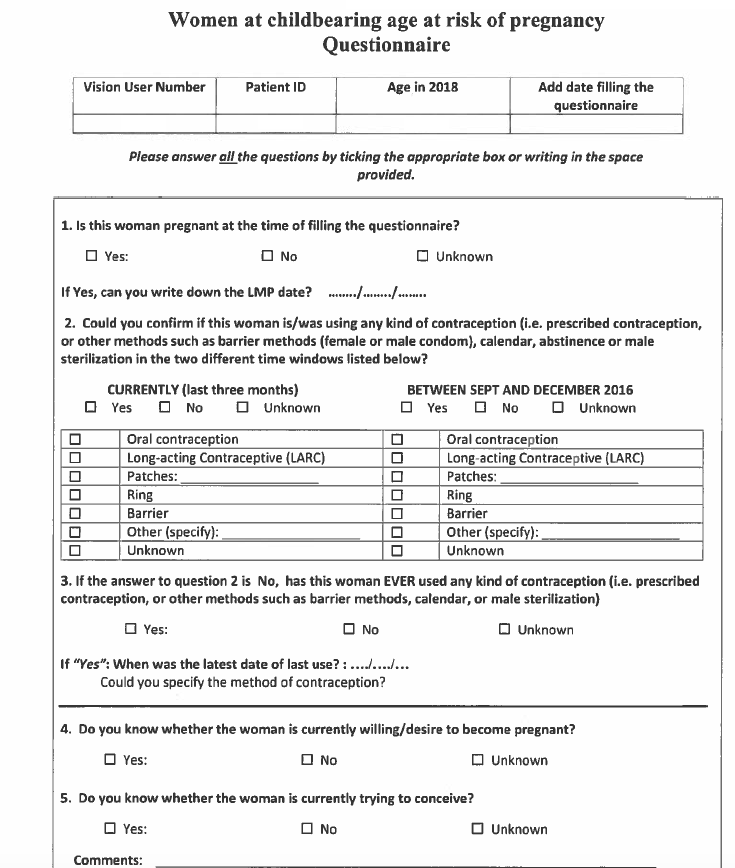
**

Supplement: Supplementary file 8 — Additional file 8. Questionnaire sent to the PCP. List of Read codes. [file 12911_2020_1184_MOESM8_ESM.docx]
